# Accelerated epigenetic aging in newborns with Down syndrome

**DOI:** 10.1111/acel.13652

**Published:** 2022-06-06

**Authors:** Keren Xu, Shaobo Li, Ivo S. Muskens, Natalina Elliott, Swe Swe Myint, Priyatama Pandey, Helen M. Hansen, Libby M. Morimoto, Alice Y. Kang, Xiaomei Ma, Catherine Metayer, Beth A. Mueller, Irene Roberts, Kyle M. Walsh, Steve Horvath, Joseph L. Wiemels, Adam J. de Smith

**Affiliations:** ^1^ Center for Genetic Epidemiology, Department of Population and Public Health Sciences Keck School of Medicine of the University of Southern California Los Angeles California USA; ^2^ Department of Paediatrics and MRC Molecular Haematology Unit, Weatherall Institute of Molecular Medicine Oxford University and BRC Blood Theme, NIHR Oxford Biomedical Centre Oxford UK; ^3^ Department of Neurological Surgery University of California San Francisco San Francisco California USA; ^4^ School of Public Health University of California, Berkeley Berkeley California USA; ^5^ Department of Chronic Disease Epidemiology Yale School of Public Health New Haven Connecticut USA; ^6^ Public Health Sciences Division, Fred Hutchinson Cancer Research Center, and Department of Epidemiology University of Washington Seattle Washington USA; ^7^ Department of Neurosurgery Duke University Durham North Carolina USA; ^8^ Department of Human Genetics David Geffen School of Medicine, University of California Los Angeles California USA

## Abstract

Accelerated aging is a hallmark of Down syndrome (DS), with adults experiencing early‐onset Alzheimer's disease and premature aging of the skin, hair, and immune and endocrine systems. Accelerated epigenetic aging has been found in the blood and brain tissue of adults with DS but when premature aging in DS begins remains unknown. We investigated whether accelerated aging in DS is already detectable in blood at birth. We assessed the association between age acceleration and DS using five epigenetic clocks in 346 newborns with DS and 567 newborns without DS using Illumina MethylationEPIC DNA methylation array data. We compared two epigenetic aging clocks (DNAmSkinBloodClock and pan‐tissue DNAmAge) and three epigenetic gestational age clocks (Haftorn, Knight, and Bohlin) between DS and non‐DS newborns using linear regression adjusting for observed age, sex, batch, deconvoluted blood cell proportions, and genetic ancestry. Targeted sequencing of *GATA1* was performed in a subset of 184 newborns with DS to identify somatic mutations associated with transient abnormal myelopoiesis. DS was significantly associated with increased DNAmSkinBloodClock (effect estimate = 0.2442, *p* < 0.0001), with an epigenetic age acceleration of 244 days in newborns with DS after adjusting for potential confounding factors (95% confidence interval: 196–292 days). We also found evidence of epigenetic age acceleration associated with somatic *GATA1* mutations among newborns with DS (*p* = 0.015). DS was not associated with epigenetic gestational age acceleration. We demonstrate that accelerated epigenetic aging in the blood of DS patients begins prenatally, with implications for the pathophysiology of immunosenescence and other aging‐related traits in DS.

## Graphical abstract

In newborns with Down syndrome (DS), epigenetic aging clocks measured in blood were significantly higher than in newborns without DS, with an epigenetic age acceleration of 244 days after adjusting for potential confounding factors. Somatic GATA1 mutations were associated with epigenetic age acceleration among newborns with DS. Accelerated epigenetic aging in DS appears to begin prenatally, with implications for the pathophysiology of immunosenescence and other aging‐related traits in DS.

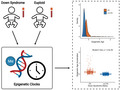

**Abbreviations**
AA: age acceleration
AD: Alzheimer’s disease
ALL: acute lymphoblastic leukemia
CBP: California Biobank Program
CCRLP: California Cancer Records Linkage Project
DBS: dried bloodspot samples
DS: Down syndrome
DS‐ALL: Down syndrome acute lymphoblastic leukemia
ML‐DS: myeloid leukemia in Down syndrome
nRBC: nucleated red blood cell
PC: principal component
QC: quality control
T21: trisomy of chromosome 21
TAM: transient abnormal myelopoiesis
VAF: variant allele fraction

## 1 INTRODUCTION

Down syndrome (DS) is the most common chromosomal disorder, affecting approximately one in every 700 babies born in the United States (Mai et al., 2019). DS is caused by constitutional trisomy of chromosome 21 (T21) and is associated with an array of phenotypes, typically including developmental delay and characteristic facial dysmorphism, and congenital heart disease in approximately 50% of individuals (Antonarakis et al., 2020). In early life, DS is also associated with defects in neonatal hematopoiesis and dysregulation of the developing immune system, and an increased risk of both lymphoid and myeloid malignancies in childhood (Hasle et al., 2000; Jardine et al., 2021; Roy et al., 2012; Verstegen et al., 2020). In adults with DS, accelerated aging is a hallmark feature that manifests phenotypically in the premature aging of the skin, hair, and immune and endocrine systems, and in early‐onset Alzheimer's disease (AD) (Devenny et al., 2005; Zigman, 2013).

Using “epigenetic clocks,” it has previously been demonstrated that accelerated epigenetic aging occurs in the blood and brain tissues of adults with DS (Horvath et al., 2015), supporting that accelerated aging may underlie senescence‐associated conditions in these tissues among individuals with DS. More recently, accelerated epigenetic aging in DS was discovered in fetal retinal cells in a small number of T21 samples (Hoshino et al., 2019), however, when the premature aging of blood cells in DS begins has yet to be examined. Here, we investigated whether accelerated epigenetic aging in DS is already detectable in whole blood samples obtained at birth, using two epigenetic clocks (pan tissue, and the skin and blood clock) that were developed with newborn blood samples in their training sets and are applicable to individuals across the lifespan.

## 2 RESULTS

Our analyses included 346 newborns with DS and 567 newborns without DS, with available neonatal dried bloodspot samples (DBS) from California or Washington State newborn screening programs (Table 1, see Section 4) (Muskens et al., 2021). Demographic characteristics were similar between DS and non‐DS newborns. Mean chronological age from conception at the time of blood sampling was significantly lower in DS (269 days) than in non‐DS newborns (276 days, *p* < 0.0001), driven by the significantly lower gestational age in DS newborns despite a longer average time between delivery and DBS collection (Table 1 and Supplemental Dataset). Newborns with DS had significantly lower birthweight (mean = 3030 g) than non‐DS newborns (mean = 3386 g, *p* < 0.0001).

**TABLE 1:** Characteristics of newborn study participants stratified by Down syndrome status (*n* = 913)

Variables	DS (*n* = 346)	Non‐DS (*n* = 567)	*p* value
ALL status (%)			<0.001
Control	199 (57.5)	437 (77.1)	
Case	147 (42.5)	130 (22.9)	
Sex (%)			0.229
Female	158 (45.9)	236 (41.6)	
Male	186 (54.1)	331 (58.4)	
Missing (%)	2 (0.6)		
Ethnicity (%)			0.819
Hispanic	190 (55.6)	321 (56.6)	
Other	56 (16.4)	84 (14.8)	
White	96 (28.1)	162 (28.6)	
Missing (%)	4 (1.2)		
Gestational age, days (mean [SD])	266.98 (17.65)	274.47 (13.93)	<0.001
Missing (%)	40 (11.6)	26 (5)	
Age at blood collection (mean [SD])	55.25 (49.74)	32.72 (17.46)	<0.001
Missing (%)	31 (9.0)		
Chronological age, days (mean [SD])	269.22 (17.58)	275.84 (13.80)	<0.001
Missing (%)	52 (15.0)	26 (5)	
Birthweight, g (mean [SD])	3029.90 (686.27)	3386.10 (541.77)	<0.001
Missing (%)	24 (6.9)		
DNAmSkinBloodClock (mean [SD])	−0.16 (0.29)	−0.40 (0.07)	<0.001
DNAmAge (mean [SD])	0.28 (0.61)	0.08 (0.17)	<0.001
Haftorn clock (mean [SD])	269.45 (12.18)	278.75 (8.95)	<0.001
Knight clock (mean [SD])	262.50 (14.87)	276.39 (10.91)	<0.001
Bohlin clock (mean [SD])	274.97 (11.44)	276.78 (8.56)	0.007
Excluding chr21 CpGs and IDOL CpGs			
DNAmSkinBloodClock (mean [SD])	−0.34 (0.22)	−0.53 (0.06)	<0.001
DNAmAge (mean [SD])	0.38 (0.61)	0.16 (0.18)	<0.001
nRBC status (%)			
High	60 (17.3)	1 (0.2)	<0.001
Not high	286 (82.7)	566 (99.8)	
*GATA1* mutation (%)			
No	154 (83.7)		
yes	30 (16.3)		
Missing (%)	162 (46.8)		
*GATA1* mutation VAF (mean [SD])	0.04 (0.15)		
Missing (%)	162 (46.8)		

*Note*: *p* values for continuous variables were calculated using the Student's *t* test and for categorical variables using the Chi‐squared test.

**FIGURE 1:**
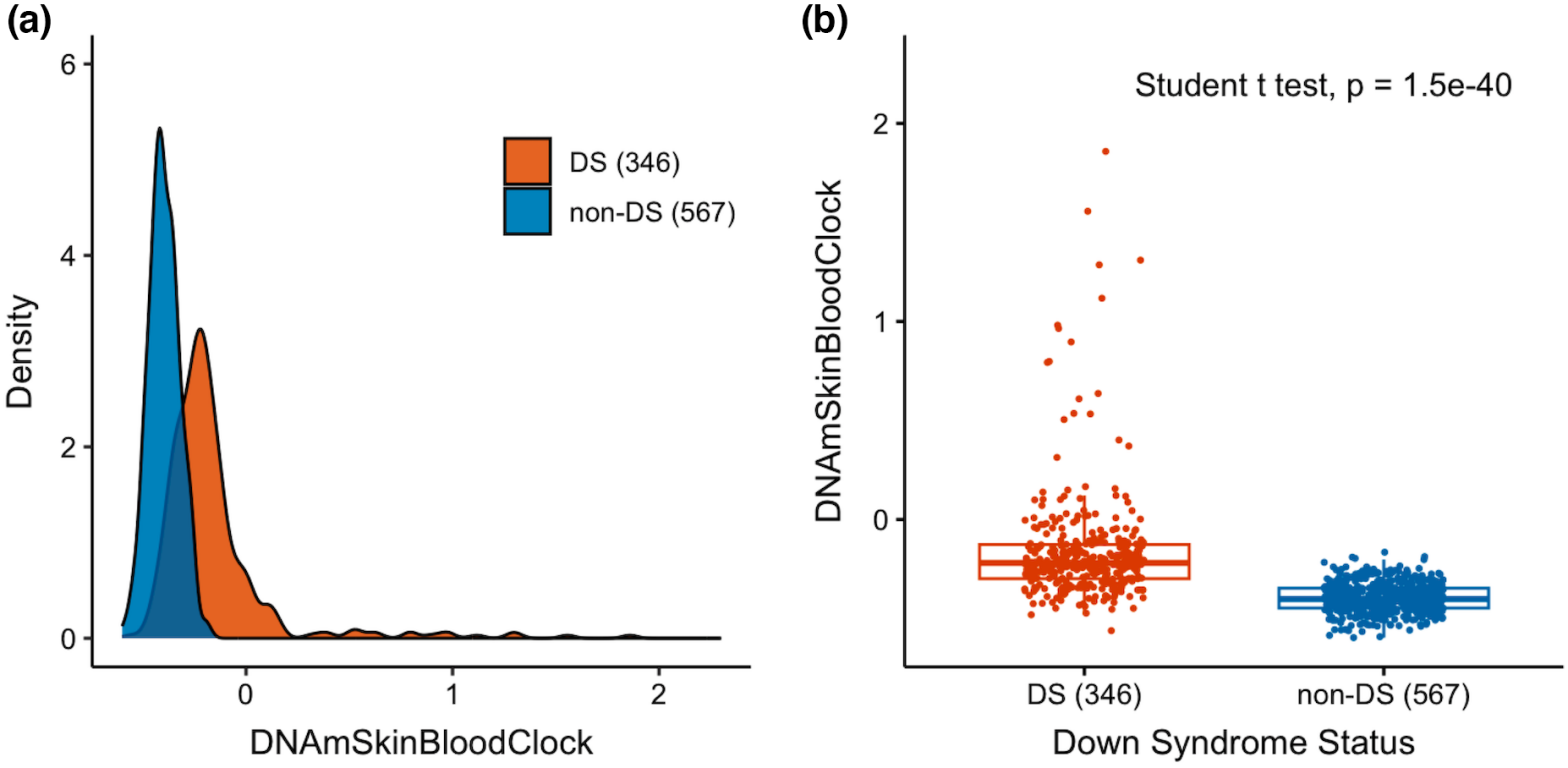
Epigenetic age in newborns with and without Down syndrome. The different distributions of the DNAmSkinBloodClock epigenetic clock in newborns with Down syndrome (DS, *n* = 346) and newborns without DS (non‐DS, *n* = 567) are shown as a density plot (panel a) and a box plot (panel b). *p* value from the Student's *t* test is shown in panel b

**FIGURE 2:**
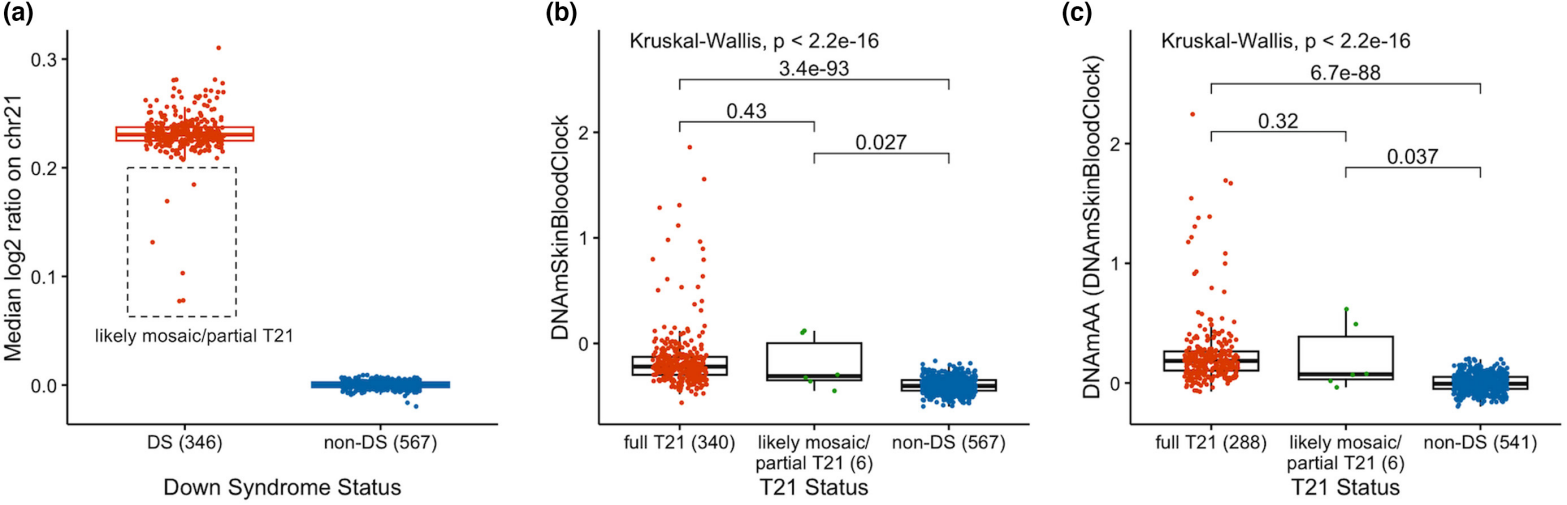
Six newborns with likely mosaic/partial trisomy 21 and their epigenetic age compared to newborns with full trisomy 21 and newborns without Down syndrome. The different distributions of the median log2 copy ratio on chromosome 21 in DS newborns (*n* = 346) and non‐DS newborns (*n* = 567) are shown as a box plot in panel a. The median log2 ratio on chromosome 21 was calculated across 317 bins generated by “conumee,” with 20 randomly selected non‐DS newborns as the reference. The 6 likely mosaic/partial DS newborns were highlighted at a median chromosome 21 log2 ratio >2 standard deviations below the average median chromosome 21 log2 ratio across all DS newborns. The different distributions of the DNAmSkinBloodClock epigenetic clock in full T21 DS newborns (*n* = 340), likely mosaic/partial T21 DS newborns (*n* = 6), and non‐DS newborns (*n* = 567) are shown as a box plot (panel b). The different distributions of the epigenetic age acceleration (DNAmAA) derived from DNAmSkinBloodClock in full T21 DS newborns (*n* = 288), likely mosaic/partial T21 DS newborns (*n* = 6), and non‐DS newborns (*n* = 541) with available birth variable data are shown as a box plot (panel c). The global *p* values from the Kruskal–Wallis test and the Benjamini–Hochberg–adjusted *p* values from the pairwise comparison tests using the Wilcoxon rank‐sum test are shown in panels b and c. Dots were overlaid on the box plot to show the individual level data colored by T21 status

Genome‐wide DNA methylation data were obtained from DBS‐derived DNA from the 346 DS and 567 non‐DS newborns using Illumina Infinium MethylationEPIC Beadchip arrays (see Section 4). We calculated two epigenetic age clocks, the pan‐tissue DNA methylation clock (DNAmAge) and the skin & blood clock (DNAmSkinBloodClock), based on the methods of Horvath (2013) and Horvath et al. (2018) (see Section 4). Bivariate tests demonstrated significantly higher DNAmSkinBloodClock (mean = −0.16 vs. −0.40, *p* < 0.0001) and DNAmAge (mean = 0.28 vs. 0.08, *p* < 0.0001) in DS newborns than in newborns without DS (Figures 1, S1A,B, Supplemental Dataset). Visual inspection of copy‐number plots generated from methylation array probe intensities revealed 6 DS newborns with median log2 ratios on chromosome 21 ranging from 0.08 to 0.18, all of which were >2 standard deviations below the average median chromosome 21 log2 ratio across all DS newborns (Figures 2a and S2). Given the low resolution and relatively low accuracy of copy‐number variant calls using DNA methylation array data (Kilaru et al., 2020), it was not possible to distinguish between mosaic and partial trisomies, thus, we termed these 6 subjects as “likely mosaic/partial T21.” Nonparametric bivariate tests showed significantly higher DNAmSkinBloodClock in the 6 likely mosaic/partial T21 newborns than in non‐DS newborns (median = −0.31 vs. −0.40, *p* = 0.027; Figure 2b). DNAmAge was similarly higher in these 6 DS newborns than in non‐DS newborns, although the result was not statistically significant (median = 0.14 vs. 0.05, *p* = 0.38; Figure S3A).

**FIGURE 3:**
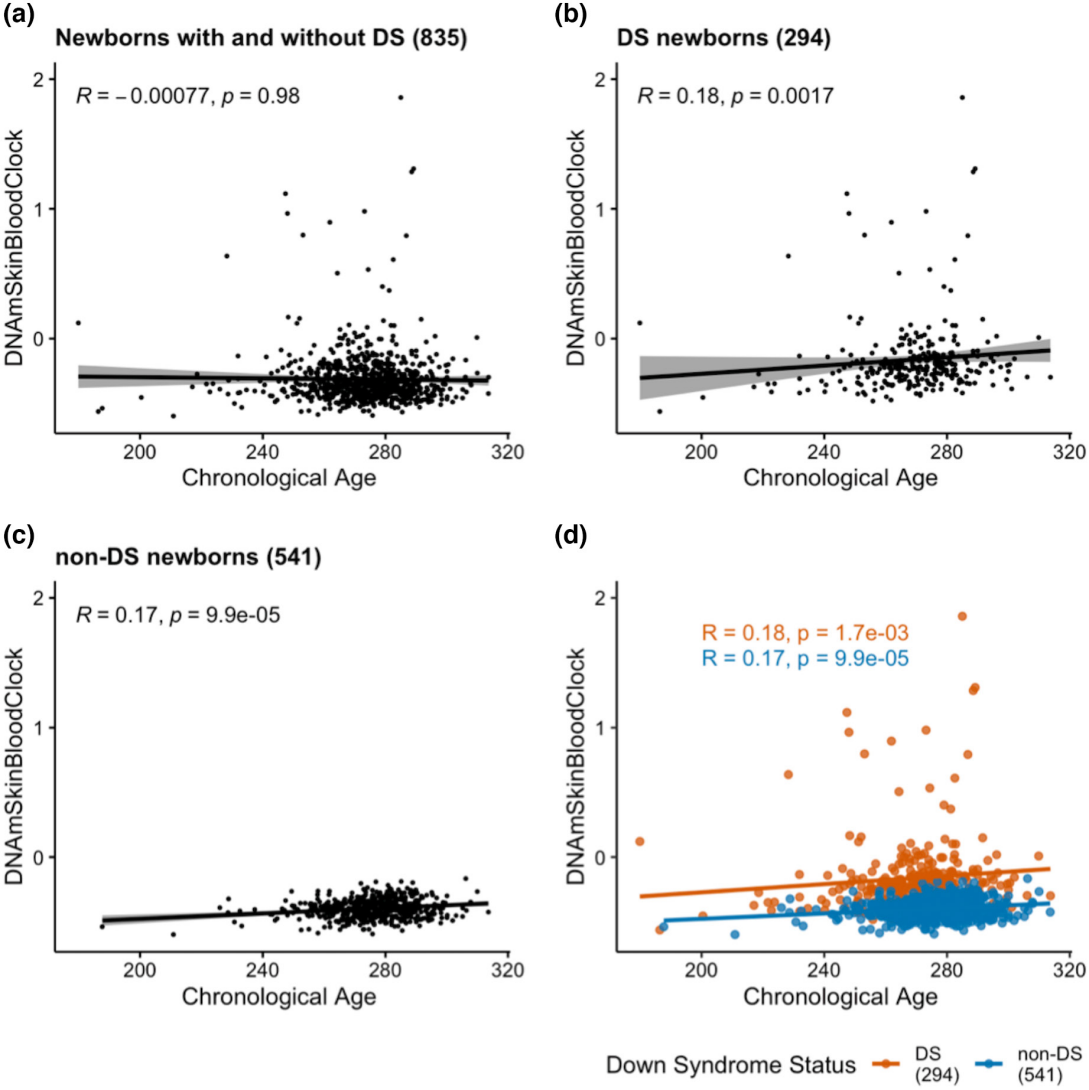
The correlations between DNAmSkinBloodClock and chronological age in newborns with and without Down syndrome. The correlations between DNAmSkinBloodClock and chronological age are shown in scatterplots for DS and non‐DS newborns combined (DS *n* = 294, non‐DS *n* = 541, panel a), for DS newborns only (*n* = 294, panel b), and for non‐DS newborns only (*n* = 541, panel c). Panel d shows the correlation between DNAmSkinBloodClock and chronological age in DS (red, *n* = 294) and in non‐DS newborns (blue, *n* = 541). Spearman correlation coefficient R and its *p* value of each correlation were summarized in panels a–d. The linear trend and its confidence interval of each correlation were summarized in panels a–c

Chronological age, calculated from gestational age plus the age at blood sampling, was significantly positively correlated with both DNAmSkinBloodClock and DNAmAge in DS (*r* = 0.18 and *r* = 0.14, respectively) and non‐DS (*r* = 0.17 and *r* = 0.15) newborns, and with similar Spearman correlation coefficients in DS compared with non‐DS subjects (Figures 3 and S1C–F). Increased DNAmSkinBloodClock was strongly correlated with increased DNAmAge in both DS (*r* = 0.62, *p* < 0.0001) and non‐DS newborns (*r* = 0.46, *p* < 0.0001) (Figure S4). Given that the DNAmSkinBloodClock had the strongest correlations with chronological age, our main results focus on this epigenetic clock.

We previously reported significant differences in deconvoluted blood cell proportions between DS and non‐DS newborns (Muskens et al., 2021). Using the same Identifying Optimal Libraries method (Gervin et al., 2019; Koestler et al., 2016), we similarly found significantly lower proportions of B lymphocytes, CD4+ T lymphocytes, granulocytes, and monocytes, and higher proportions of CD8+ T lymphocytes, natural killer cells, and nucleated red blood cells (nRBCs) in DS newborns than in non‐DS newborns in this expanded dataset, and in the additional 150 DS and 132 non‐DS subjects that were not analyzed previously (Table S1). Next, we assessed the correlations between blood cell proportions and epigenetic age (DNAmSkinBloodClock) in DS and non‐DS newborns separately, given the differences in blood cell proportions between the two groups. Epigenetic age showed a significant negative correlation with proportions of B‐cells and granulocytes in both DS and non‐DS newborns, and a significant positive correlation with nRBCs in DS newborns but not in non‐DS newborns (Figure S5). Given the differences in deconvoluted blood cell proportions between DS and non‐DS newborns and their associations with epigenetic age, we include blood cell proportions as covariates in regression models analyzing the association between epigenetic aging and DS.

**FIGURE 4:**
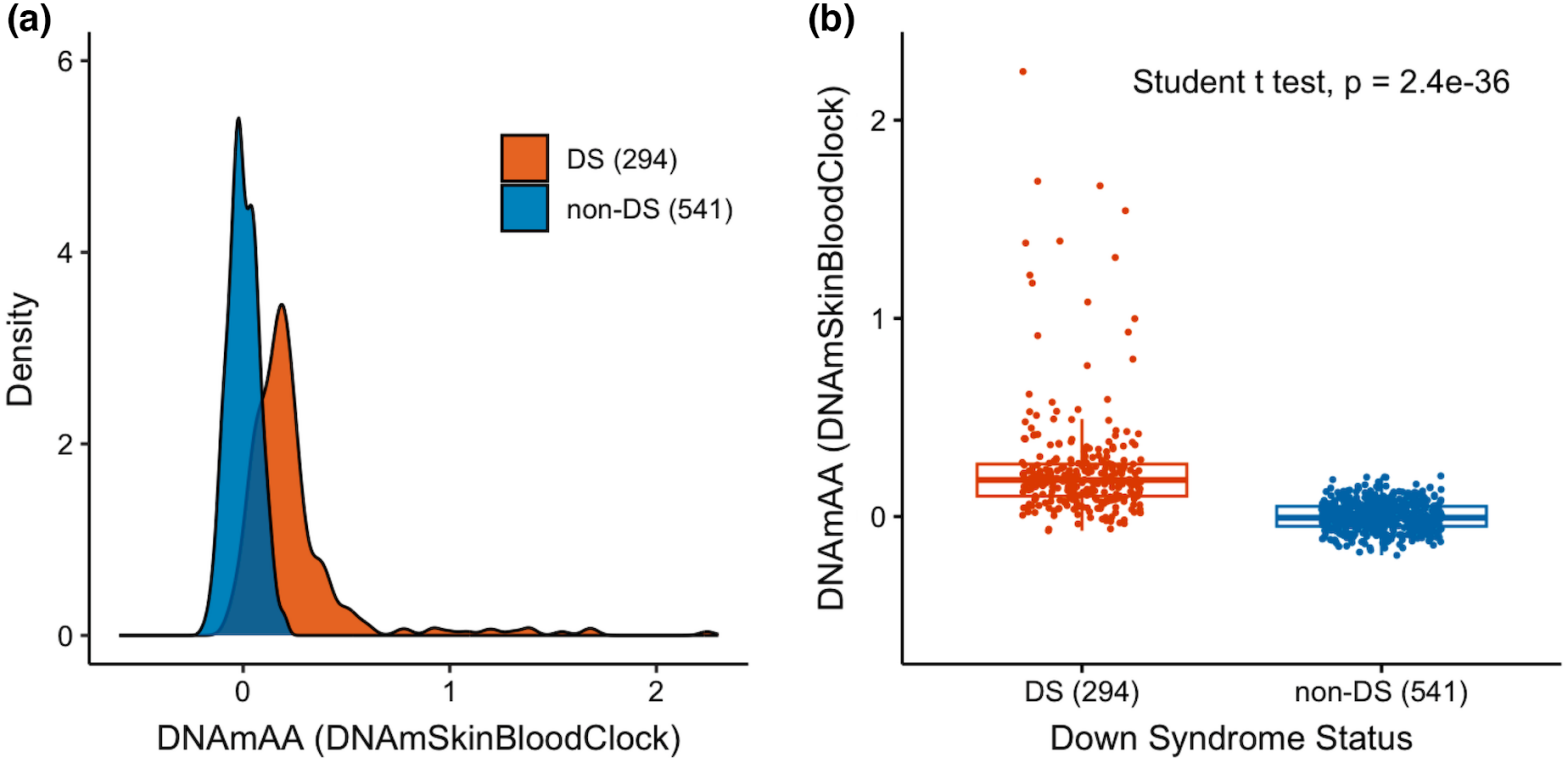
Epigenetic age acceleration in newborns with and without Down syndrome. The different distributions of DNAmAA (age acceleration using DNAmSkinBloodClock) in DS newborns (*n* = 294) and non‐DS newborns (*n* = 541) are shown as a density plot (panel a) and a box plot (panel b). *p* value from the Student's *t* test is shown in panel b

Linear regression analysis showed that each one‐day increase in chronological age corresponded to a 0.001 unit increase in DNAmSkinBloodClock in non‐DS newborns (Figure 3D). Epigenetic age of DS newborns appeared to lie above the regression line estimating the association between DNAmSkinBloodClock and chronological age in non‐DS newborns (Figure 3D), indicating that DS subjects exhibited accelerated aging effects. Thereby, we calculated epigenetic age acceleration (DNAmAA) for each subject as the deviation from the expected epigenetic age clock based on its linear association with chronological age in non‐DS newborns (the distance from the observed epigenetic age to the blue regression line of non‐DS newborns in Figure 3D). By definition, the mean epigenetic age acceleration in non‐DS newborns was zero. Mean DNAmAA was 0.2418 in DS newborns, which was significantly higher than zero (*p* < 0.0001; Figure 4). Epigenetic age acceleration derived from the pan‐tissue DNAmAge clock was also significantly different between DS and non‐DS newborns (*p* < 0.0001; Figure S1G,H). For the 6 DS newborns with likely mosaic/partial T21, we found significant epigenetic age acceleration derived from DNAmSkinBloodClock (DNAmAA median = 0.07 vs. −0.01, *p* = 0.037; Figure 2C) but not from the pan‐tissue DNAmAge clock (median = 0.04 vs. −0.02, *p* = 0.50; Figure S3B).

In linear regression models adjusting for sex, chronological age, birthweight, batch, the first 9 EPISTRUCTURE principal components (PCs), and deconvoluted blood cell‐type proportions (see Section 4), DS remained significantly associated with epigenetic aging (effect estimate = 0.2442, *p* < 0.0001), with an age acceleration of 244 days (Table 2). Here, the age acceleration was the effect estimate divided by 0.001, the increase in DNAmSkinBloodClock for every one‐day increase of chronological age in non‐DS newborns (Figure 3D).

**TABLE 2:** Association between Down syndrome and epigenetic aging and epigenetic age acceleration, in newborn blood samples using the DNAmSkinBloodClock

Model (*n* subjects)	DNAmSkinBloodClock			DNAmAA for DNAmSkinBloodClock		
	Estimate (95% CI)	*p* value	AA for DS (days)	Estimate (95% CI)	*p* value	AA for DS (days)
Base model (834)	0.3477 (0.3097–0.3858)	8.03 × 10^−61^	347.7	0.3497 (0.3119–0.3875)	4.51 × 10^−62^	349.7
Full model (834)	0.2442 (0.1964–0.2920)	2.54 × 10^−22^	244.2	0.2406 (0.1933–0.2880)	3.91 × 10^−22^	240.6
Full model^a^ (783)	0.1322 (0.0958–0.1685)	2.35 × 10^−12^	132.2	0.1282 (0.0921–0.1642)	6.99 × 10^−12^	128.2
Full model^b^ (680)	0.1730 (0.1244–0.2216)	7.55 × 10^−12^	173.0	0.1735 (0.1251–0.2219)	5.34 × 10^−12^	173.5

*Note*: Base model is linear regression for DNAmSkinBloodClock or DNAmAA (DNAmSkinBloodClock) as a function of DS status, adjusting for sex, chronological age (chronological age was not adjusted for DNAmAA), birth weight, EPISTRUCTURE PCs (9 PCs in the model for DNAmSkinBloodClock and 10 PCs in the model for DNAmAA), and batch; full model additionally adjusted for blood cell proportions.

Abbreviation: AA, age acceleration.

^a^ Full model in subjects without high nRBC.

^b^ Full model in DS newborns that were found to be *GATA1* mutation wildtype and non‐DS newborns.

A subset of DS newborns (*N* = 60, 17.3%) and one (0.2%) non‐DS newborn had markedly high levels (>25%) of deconvoluted nRBC proportions, which we recently demonstrated has a significant influence on global patterns of DNA methylation (Muskens et al., 2021). Thus, we repeated the age acceleration analysis excluding the high nRBC newborns, and DS remained significantly associated with epigenetic aging (effect estimate = 0.1322, *p* < 0.0001, age acceleration = 132 days). We performed an additional sensitivity analysis to account for the potential confounding effect of transient abnormal myelopoiesis (TAM) or silent TAM, which occur in up to 30% of newborns with DS and are driven by somatic mutations in the *GATA1* gene (Roberts et al., 2013). Limiting our regression analysis to the 139/164 DS newborns that were found to be *GATA1* mutation wildtype (and hence would not have TAM/Silent TAM) by targeted sequencing and with all covariates available (Muskens et al., 2021) compared with non‐DS newborns, we found that DS remained associated with epigenetic aging (effect estimate = 0.1730, *p* < 0.0001, age acceleration = 173 days) (Table 2).

We further attempted to assess the association between DS and epigenetic age acceleration directly. We fitted a linear regression model estimating DNAmAA as a function of DS status, sex, birthweight, batch, cell‐type proportions, and the first 10 EPISTRUCTURE PCs. DS was significantly associated with increased DNAmAA (effect estimate = 0.2406, *p* < 0.0001) (Table 2); the associations remained after excluding the high nRBC newborns (effect estimate = 0.1282, *p* < 0.0001) and when limited to *GATA1*‐wildtype DS newborns (effect estimate = 0.1735, *p* < 0.0001). An additional sensitivity analysis was performed to account for potential confounding of CpGs on the trisomic chromosome 21, or those associated with blood cell proportions, in calculation of the epigenetic clock. We recalculated DNAmBloodSkinClock and DNAmAA excluding CpG sites on chromosome 21 and CpGs used in the reference set for the cell‐type deconvolution, and DS remained significantly associated with the epigenetic clock (effect estimate = 0.1987, *p* < 0.0001) and with DNAmAA (effect estimate = 0.1959, *p* < 0.0001) (Table S2). Taken together, these data support that accelerated epigenetic aging is already detectable at birth in the whole blood of individuals with DS.

Children with DS have a markedly high risk of hematological malignancy (Hasle et al., 2000). Our study included a large proportion of children that went on to develop ALL (Table 1), providing an opportunity to explore whether epigenetic age acceleration at birth in newborns with DS may be associated with increased risk of ALL in childhood (DS‐ALL); however, we found no significant difference in epigenetic age or DNAmAA between DS‐ALL subjects (*N* = 147) and DS non‐ALL subjects (*N* = 199) (Table S3 and Figure S6).

Myeloid leukemia in DS (ML‐DS) cases were not included in our study, but we were able to investigate whether epigenetic age may be associated with somatic *GATA1* mutations at birth, which can progress to ML‐DS in up to 10% of patients (Roberts et al., 2013). We compared epigenetic age estimates in newborns with DS with (*n* = 30) and without (*n* = 154) *GATA1* mutations, as assessed by targeted sequencing (see Section 4), using both bivariate tests and linear regression models adjusting for cell‐type proportions (Figure S7 and Table S4). We found that the presence of *GATA1* mutations at birth was associated with increased epigenetic age (effect estimate = 0.1441, *p* = 0.015), with an age acceleration of 144 days in *GATA1* mutation‐positive newborns (Table S4). Further, *GATA1* mutation variant allele fraction (VAF) was positively associated with epigenetic age among mutation‐positive newborns (effect estimate = 1.7805, *p* = 0.004), with an age acceleration of 178 days per 10% increase in *GATA1* mutation VAF. *GATA1* mutations were also significantly associated with increased DNAmAA, both as a binary variable (effect estimate = 0.1447, *p* = 0.009) and with increasing mutation VAF (effect estimate = 1.5390, *p* = 0.016). In sensitivity analyses, *GATA1* mutations and mutation VAF remained significantly associated with the epigenetic clock and DNAmAA after excluding CpGs on chromosome 21 or in the blood cell deconvolution references (Table S5).

Finally, we explored whether newborns with DS may also demonstrate accelerated epigenetic gestational aging, using three gestational age clocks (Haftorn, Knight, and Bohlin, see Section 4) previously developed using genome‐wide DNA methylation data (Bohlin et al., 2016; Haftorn et al., 2021; Knight et al., 2016). The three gestational age clocks were significantly positively associated with each other in DS and non‐DS newborns, with correlation coefficients ranging from 0.72 to 0.93 (Figure S8). They were also significantly positively associated with the observed gestational age in both DS (correlation coefficient ranges: 0.46–0.59) and non‐DS newborns (correlation coefficient ranges: 0.49–0.51) (Figure S9). Mean observed gestational age was significantly lower in DS (267 days) than in non‐DS newborns (274 days, *p* < 0.0001) (Table 1, Figure S10A,B). Bivariate tests also showed significantly lower gestational age clocks in DS newborns than in newborns without DS (*p*_Haftorn_ < 0.0001, *p*_Knight_ < 0.0001, *p*_Bohlin_ = 0.012) (Table 1, Figure S10C–H). DS remained significantly associated with lower gestational age clocks in linear regression models adjusting for observed gestational age, sex, batch, birthweight, cell‐type proportions, and EPISTRUCTURE PCs (Table S6).

To evaluate epigenetic gestational age acceleration, we derived DNAmAA from each gestational age clock. For all three clocks, gestational age acceleration was not significantly different between DS and non‐DS newborns in bivariate tests or in linear regression models adjusting for sex, batch, birthweight, cell‐type proportions, and EPISTRUCTURE PCs (Figure S11, Table S7). These results contrast our findings for the epigenetic age clock analyses and suggest that there is no significant gestational age acceleration in newborns with DS.

## 3 DISCUSSION

In summary, we performed the largest study of epigenetic aging in DS to date, and discovered significant accelerated epigenetic aging in the whole blood of newborns with DS, supporting that accelerating aging begins early in life in individuals with DS. We found an average 244‐day acceleration in epigenetic aging among newborns with DS after adjusting for potential confounding factors, which was reduced to 132 days after excluding the subset of (mainly DS) newborns with high nRBC proportions. The causes of premature aging in DS remain to be determined but may result from genome‐wide perturbations in epigenetic regulation and gene expression associated with T21 (Lane et al., 2014; Letourneau et al., 2014; Muskens et al., 2021) and are potentially linked to the upregulation of inflammatory processes in individuals with DS (Huggard et al., 2020; Sullivan et al., 2017), which is further supported by the differences in hematopoiesis revealed by our cell‐type deconvolution analyses. Understanding the mechanisms involved may reveal novel therapeutic targets to help ameliorate the progeroid features of DS.

It is also important to investigate whether differences in premature aging may underlie phenotypic variation in individuals with DS. We found no association between accelerated epigenetic aging at birth and future risk of DS‐ALL development. We did, however, find that DS newborns harboring somatic *GATA1* mutations have increased epigenetic aging relative to DS newborns without *GATA1* mutations. Given the pathophysiology of TAM/Silent TAM, it is possible that epigenetic age acceleration was the consequence of the *GATA1* mutation phenotype rather than a risk factor, and there may have also been residual confounding by the increased nRBC proportions in DS newborns with *GATA1* mutations ^11^. Future studies will be required to assess the role of epigenetic aging in the risk of ML‐DS and in adult‐onset neurologic traits like DS‐AD.

The lack of accelerated epigenetic gestational age in newborns with DS suggests that the epigenetic clocks for gestational age may be specific to the timing of factors related to gestation, such as fetal growth and developmental maturation, rather than general processes of aging (Haftorn et al., 2021; Knight et al., 2016). DS is associated with fetal growth restriction and an increased frequency of small‐for‐gestational‐age births compared with non‐DS newborns (Muskens et al., 2021; Yao et al., 2020), which may explain the significantly lower epigenetic gestational age clocks in DS newborns in our study, even after adjusting for observed gestational age. Whereas CpGs most informative for fetal developmental profiling gave qualitatively different results than the DNAmSkinBloodClock and DNAmAge clock, these latter clocks incorporate the CpGs most informative for predicting aging across the life course. The substantial aging acceleration observed in DS newborns using these clocks likely reflects the fetal programming of longer‐term somatic aging, which appears to be initiated much earlier in the DS population.

The strengths of our study include the use of DBS samples for a large number of newborns with and without DS, the availability of both gestational age and age at DBS collection that enabled us to calculate chronological age from conception, and the inclusion of DS‐ALL cases and *GATA1* mutation data that allowed us to explore the association between epigenetic age and hematological phenotypes in DS. The exclusive use of newborn DBS, however, limited our ability to assess epigenetic aging in DS outside of the neonatal period, although previous studies have demonstrated accelerated epigenetic aging in older DS individuals (Horvath et al., 2015). The usage of archived neonatal dried blood spots might have affected the DNA methylation measurements; however, this biospecimen type has been shown to provide high‐quality DNA for methylation arrays (Hollegaard et al., 2013; Walker et al., 2019). Phenotypic information was also limited for subjects in our study, and it is possible that some unmeasured DS‐related conditions may have affected our results. Understanding the effects of premature aging on common DS‐related phenotypes will require longitudinal studies with an assessment of epigenetic clocks and other aging‐related biomarkers in well‐phenotyped individuals with and without DS and follow‐up throughout the life course.

## 4 EXPERIMENTAL PROCEDURES

### 4.1 Study subjects

This study was approved by Institutional Review Boards at the California Health and Human Services Agency, University of Southern California, University of Berkeley, Yale University, and Washington State.

Archived neonatal dried bloodspot (DBS) specimens were obtained from 351 newborns with DS and 574 newborns without DS. Of the 351 subjects with DS, DBS for 198 newborns were obtained from the California Biobank Program (CBP) as previously described (Muskens et al., 2021). In brief, these 198 DS newborns did not have a leukemia diagnosis by the age of 15 years (DS non‐ALL), as identified via linkage between the California Department of Public Health Genetic Disease Screening Program and the California Cancer Registry (Wiemels et al., 2018). An additional 152 DS newborns who later developed ALL (DS‐ALL) were included in the International Study of Down Syndrome Acute Leukemia (Brown et al., 2019), including 114 identified in the California Cancer Records Linkage Project (CCRLP) (Wiemels et al., 2018), 19 in the California Childhood Leukemia Study (Metayer et al., 2013), and 19 identified in the Washington State Childhood Cancer study using population‐based linked birth‐hospital discharge‐cancer registry records and with DBS obtained from the Washington State Department of Health Newborn Screening Program (Mueller et al., 2018). One additional DS non‐ALL subject was identified in the CCRLP.

Of the 574 newborns without DS, these included 133 non‐DS ALL cases born in California and 441 non‐DS and cancer‐free control children who were matched to cases by year and county of birth (3 or 4 controls per case), as previously described (Nielsen et al., 2019). Cancer diagnosis data were obtained from the California Cancer Registry and neonatal DBS from the CBP. Birth‐related variables including birthweight, gestational age, and age at DBS collection (age at blood sampling) were collected for the majority of California‐born subjects from the CBP (Table 1).

### 4.2 DNA methylation arrays

DNA was isolated from one‐third portions of each DBS using the Qiagen DNA Investigator blood card protocol. DNA samples were bisulfite‐converted using Zymo EZ DNA Methylation kits, with total DNA inputs ranging from 115 to 451 ng as measured by PicoGreen. Bisulfite‐converted DNA was then assayed on Illumina Infinium MethylationEPIC Beadchip genome‐wide DNA methylation arrays. DS and non‐DS samples were block‐randomized to ensure equivalent distributions of sex, self‐reported race/ethnicity, and ALL case–control status, on all plates.

### 4.3 DNA methylation array data processing

Initial quality control (QC) of MethylationEPIC array data was performed using Illumina GenomeStudio Software and BeadArray Controls Reporter Software, including evaluation of control probes for bisulfite treatment conversion efficiency, dye specificity, hybridization, and staining, and control probes performed robustly for all samples supporting the generation of high‐quality data. Ten intra‐plate duplicates, with median DNA inputs of 201 ng (range: 115 to 451 ng), showed a strong correlation of methylation beta values with *R* ^2^ ≥ 0.995. One DS sample was excluded based on a mismatch between reported sex and methylation gender controls data. Additional QC assessment and normalization of DNA methylation array data were performed using the “minfi” package (Aryee et al., 2014) through the Bioconductor project (Gentleman et al., 2004; Huber et al., 2015). Functional normalization (Funnorm) was performed using the “preprocessFunnorm” function (Fortin et al., 2014) to remove technical variability and batch effects. By default, the “preprocessFunnorm” function applies the noob within‐array normalization as the first step to correct for background fluorescence and dye bias. Funnorm, together with noob, has been found to outperform the individual normalization methods and other batch removal tools (Fortin et al., 2014). Liu et al. reported that Funnorm/noob plus beta‐mixture quantile normalization (BMIQ) improved signal sensitivity and reduced technical variance (Liu & Siegmund, 2016). Therefore, the BMIQ method was subsequently applied (Teschendorff et al., 2013). Mean detection *p* values were calculated by using the “detectionP” function (Aryee et al., 2014). CpGs with mean detection *p* value >0.01 were considered poor quality and were removed from the analysis. CpG sites and samples that had missingness >15% were removed. Four DS and 7 non‐DS subjects were excluded after QC. To confirm constitutive T21 status, we generated copy‐number variation plots using the R package “conumee” (Mah et al., 2018) for all subjects, with 20 randomly selected non‐DS newborns as the reference. Median log2 copy‐number ratios on chromosome 21 were calculated across 317 bins for all the newborns with and without DS, with values ≥0.2 in DS newborns indicating full T21 status. Samples with a median chromosome 21 log2 copy ratio that was >2 standard deviations below the average median chromosome 21 log2 copy ratio across all DS newborns, but above the maximum median chromosome 21 log2 copy ratio of non‐DS newborns, were designated as likely mosaic/partial T21.

### 4.4 Epigenetic age clock calculations

DNA methylation‐based estimators of age were calculated according to the methods from Horvath (2013) and Horvath et al. (2018). We computed two epigenetic age clocks: the pan‐tissue DNA methylation clock (DNAmAge) and the skin & blood clock (DNAmSkinBloodClock). Briefly, a calibrated version of DNAmAge was calculated from a weighted combination of the DNA methylation levels of 353 CpGs and was subsequently converted back to DNAmAge; DNAmSkinBloodClock was calculated in a similar way from 391 CpGs. Of the 353 clock CpGs comprising DNAmAge, 19 were missing from the EPIC array because they were only present on the Illumina Infinium 450 K and 27 K arrays used by the original training set. The missing values were replaced with the average beta values of the available clock CpGs (*n* = 334) per sample using the “impute.knn” function from the “impute” package (Hastie et al., 2020). The two epigenetic age clocks share 60 CpGs. Two age‐independent measures of epigenetic age acceleration (DNAmAA) were derived from DNAmAge and DNAmSkinBloodClock based on the methods from Horvath et al. (2015) DNAmAA was calculated as the deviation from the expected epigenetic age clock based on its linear association with chronological age (gestational age plus age at blood sampling) in non‐DS newborns.

### 4.5 Gestational age clock calculations

We calculated three epigenetic gestational age clocks using the approaches as previously described (Bohlin et al., 2016; Haftorn et al., 2021; Knight et al., 2016). The Haftorn clock (Haftorn et al., 2021) comprises 176 CpGs on the EPIC array. The Knight clock (Knight et al., 2016) and the Bohlin clock (Bohlin et al., 2016) are composed of 148 CpGs and 96 CpGs from the 450K array, respectively. Six CpGs in the Knight clock and 8 CpGs in the Bohlin clock were absent from the EPIC array and were thus replaced with the average beta values of the 142 and 88 available clock CpGs, respectively, using the “impute.knn” function. The number of the CpGs overlapping each pair of the epigenetic clocks is summarized in Figure S12. DNAmAA for each epigenetic gestational age clock was calculated as the residuals from the linear association of each gestational age clock with the observed gestational age in newborns adjusted for DS status.

### 4.6 Assessment of cell‐type heterogeneity

We performed reference‐based deconvolution of blood cell proportions in all subjects using the Identifying Optimal Libraries algorithm (Gervin et al., 2019; Koestler et al., 2016). Proportions of monocytes, granulocytes, natural killer cells, B lymphocytes, T lymphocytes (both CD4+ and CD8+), and nucleated red blood cell (nRBC)/erythroblasts were estimated by using the “estimateCellCounts2” function in the R package “FlowSorted.Blood.EPIC” (Salas & Koestler, 2021) coupled with DNA methylation data from cord blood cell reference samples in the R package “FlowSorted.CordBloodCombined.450k” (Bakulski et al., 2016).

### 4.7 *GATA1* sequencing

Targeted sequencing of *GATA1* exons 2 and 3 was performed in a subset of 184 newborns with DS who did not develop leukemia, as previously described (Labuhn et al., 2019; Muskens et al., 2021; Roberts et al., 2013), to identify DS newborns with preleukemia associated with transient abnormal myelopoiesis (TAM) or silent TAM.

### 4.8 Statistical analysis

All statistical analyses were performed in R v 4.0.2 (R Core Team, 2020) with significance tests using 2‐sided *p* = 0.05. Means and standard deviations were summarized for continuous variables, including birthweight, gestational age, age at blood sampling, chronological age from conception (gestational age plus age at blood sampling), and epigenetic estimators of age. Frequencies and proportions were computed for categorical variables (sex, self‐reported race/ethnicity, and ALL case/control status). We compared continuous variables and categorical variables between DS newborns and non‐DS newborns using the Student's *t* test and *χ* ^2^, respectively. We also tested for the differences in epigenetic age and epigenetic age acceleration between DS newborns with full T21, with likely mosaic/partial T21 and non‐DS newborns using the nonparametric Kruskal–Wallis test. The Benjamini–Hochberg adjusted *p* values were subsequently obtained from the Wilcoxon rank‐sum pairwise comparison tests.

We computed Spearman correlation coefficients for DNAmSkinBloodClock, DNAmAge, and chronological age from conception, and for the three gestational age clocks with the observed gestational age, in DS and non‐DS newborns. We also tested the association of cell‐type proportions with epigenetic estimators using the Spearman correlation test. In addition, we fit separate linear regression models with each blood cell‐type proportion as the dependent variable and DS status as the independent variable, adjusting for sex, gestational age, age at DBS collection, birthweight, batch, and genetic ancestry using the first 10 PCs derived from EPISTRUCTURE (Rahmani et al., 2017).

We further compared the epigenetic clocks between DS and non‐DS newborns using linear regression adjusting for chronological age from conception, sex, birthweight, batch, cell‐type proportions, and genetic ancestry using PCs derived from EPISTRUCTURE. The linear regression models for the gestational age clocks were adjusted for gestational age instead of chronological age. In addition, we compared age‐independent measures of epigenetic age acceleration between DS and non‐DS newborns using linear regression models adjusting for the same covariates described above, excluding chronological age or gestational age. We adjusted for the covariates that had *p* values <0.2 from the univariable linear regression analyses for epigenetic estimators in non‐DS newborns. Likelihood ratio tests and Akaike's Information Criteria were used to determine how many EPISTRUCTURE PCs should be included in the regression models. Six out of 7 blood cell proportions were adjusted in the linear regression models; the cell proportion of granulocytes was excluded from the models to avoid multicollinearity. DNAmSkinBloodClock and DNAmAA were additionally compared between DS newborns who later developed ALL and DS newborns without ALL using bivariate tests and linear regression models.

We repeated analyses excluding 61 newborns (60 DS) with nRBC proportions exceeding 25% or limiting to DS newborns that were found to be *GATA1* mutation wildtype. In addition, among DS newborns with available *GATA1* sequencing, we tested for associations of *GATA1* mutation status and VAF with epigenetic age estimators, using linear regression adjusting for the same covariates described above.

Finally, we recalculated DNAmSkinBloodClock excluding CpG sites on chromosome 21 or CpGs used in the reference set for the deconvolution of blood cell proportions (10/391) and repeated all linear regression analyses to address potential confounding.

## AUTHOR CONTRIBUTIONS

Adam J. de Smith and Joseph L. Wiemels designed and supervised this study. Keren Xu, Shaobo Li, Ivo S. Muskens, Natalina Elliott, Priyatama Pandey, and Adam J. de Smith analyzed the data. Natalina Elliott, Swe Swe Myint, and Helen M. Hansen performed the experiments. Libby M. Morimoto, Alice Y. Kang, Xiaomei Ma, Catherine Metayer, and Beth A. Mueller provided the resources. Keren Xu, Kyle M. Walsh, Steve Horvath, Joseph L. Wiemels, and Adam J. de Smith prepared the manuscript. All authors edited and approved the paper.

## CONFLICT OF INTEREST

The authors declare that they have no conflict of interest.

## Supporting information


**FIGURE S1** DNAmAge (pan‐tissue clock) and age acceleration in newborns with and without Down syndrome
**FIGURE S2** Six newborns with Down syndrome with likely mosaic/partial trisomy 21
**FIGURE S3** DNAmAge (pan‐tissue clock) and age acceleration in newborns with Down syndrome with full trisomy 21, with likely mosaic/partial trisomy 21 and in newborns without Down syndrome
**FIGURE S4** The correlation between DNAmSkinBloodClock and DNAmAge
**FIGURE S5** The correlations between the blood cell proportions and DNAmSkinBloodClock in DS (red, *n* = 346) and non‐DS (blue, *n* = 567) newborns
**FIGURE S6** Epigenetic age and age acceleration in Down syndrome newborns with and without acute lymphoblastic leukemia
**FIGURE S7** Epigenetic age and age acceleration in Down syndrome newborns with *GATA1* mutation and without *GATA1* mutation
**FIGURE S8** The correlations between the Haftorn, Knight, and Bohlin clocks in DS and non‐DS newborns combined (*n* = 913), in DS newborns (*n* = 346), and in non‐DS newborns (*n* = 567)
**FIGURE S9** The correlations between the three epigenetic gestational age clocks and the observed gestational age in DS and non‐DS newborns combined (*n* = 847), in DS newborns (*n* = 306), and in non‐DS newborns (*n* = 541)
**FIGURE S10** The observed gestational age and the three gestational epigenetic age clocks in newborns with and without Down syndrome
**FIGURE S11** DNAmAA derived from the three epigenetic gestational age clocks in DS newborns (*n* = 306) and non‐DS newborns (*n* = 541)
**FIGURE S12** The number of the CpGs overlapping each pair of the epigenetic clocks (i.e., DNAmSkinBloodClock, DNAmAge, Haftorn clock, Knight clock, and Bohlin clock)

**TABLE S1** Differences in the deconvoluted blood cell proportions between newborns with and without Down syndrome
**TABLE S2** Associations between Down syndrome and epigenetic aging, using modified epigenetic clock excluding chromosome 21 CpGs or CpGs used in the reference set for the deconvolution of blood cell proportion
**TABLE S3** Associations between DS‐ALL and epigenetic aging among newborns with Down syndrome
**TABLE S4** Association between somatic GATA1 mutations and epigenetic aging and epigenetic age acceleration, in blood samples among newborns with Down syndrome
**TABLE S5** Association between somatic GATA1 mutations and epigenetic aging and epigenetic age acceleration, in blood samples among newborns with Down syndrome, using modified epigenetic clock excluding chromosome 21 CpGs or CpGs used in the reference set for the deconvolution of blood cell proportion
**TABLE S6** Associations between Down syndrome and three epigenetic gestational age clocks
**TABLE S7** Associations between Down syndrome and epigenetic gestational age acceleration (DNAmAA) derived from three epigenetic gestational age clocks

DATA S1

## Data Availability

This study used biospecimens from the California Biobank Program. Any uploading of genomic data (including genome‐wide DNA methylation data) and/or sharing of these biospecimens or individual data derived from these biospecimens has been determined to violate the statutory scheme of the California Health and Safety Code Sections 124980(j), 124991(b), (g), (h), and 103850 (a) and (d), which protect the confidential nature of biospecimens and individual data derived from biospecimens. Data for epigenetic clocks and available covariates in newborns with and without DS are included in the Supplemental Dataset.
