# Supplementary material for: Accelerated epigenetic aging in newborns with Down syndrome
Source: Aging Cell. 2022 Jun 6;21(7):e13652. doi: 10.1111/acel.13652 (PMC9282838; doi:10.1111/acel.13652)
Supplement: Supplementary file 1 — FIGURE S1 DNAmAge (pan‐tissue clock) and age acceleration in newborns with and without Down syndrome FIGURE S2 Six newborns with Down syndrome with likely mosaic/partial trisomy 21 FIGURE S3 DNAmAge (pan‐tissue clock) and age acceleration in newborns with Down syndrome with full trisomy 21, with likely mosaic/partial trisomy 21 and in newborns without Down syndrome FIGURE S4 The correlation between DNAmSkinBloodClock and DNAmAge FIGURE S5 The correlations between the blood cell proportions and DNAmSkinBloodClock in DS (red, n = 346) and non‐DS (blue, n = 567) newborns FIGURE S6 Epigenetic age and age acceleration in Down syndrome newborns with and without acute lymphoblastic leukemia FIGURE S7 Epigenetic age and age acceleration in Down syndrome newborns with GATA1 mutation and without GATA1 mutation FIGURE S8 The correlations between the Haftorn, Knight, and Bohlin clocks in DS and non‐DS newborns combined (n = 913), in DS newborns (n = 346), and in non‐DS newborns (n = 567) FIGURE S9 The correlations between the three epigenetic gestational age clocks and the observed gestational age in DS and non‐DS newborns combined (n = 847), in DS newborns (n = 306), and in non‐DS newborns (n = 541) FIGURE S10 The observed gestational age and the three gestational epigenetic age clocks in newborns with and without Down syndrome FIGURE S11 DNAmAA derived from the three epigenetic gestational age clocks in DS newborns (n = 306) and non‐DS newborns (n = 541) FIGURE S12 The number of the CpGs overlapping each pair of the epigenetic clocks (i.e., DNAmSkinBloodClock, DNAmAge, Haftorn clock, Knight clock, and Bohlin clock) [file ACEL-21-e13652-s002.pdf]

Accelerated Epigenetic Aging in Newborns with Down Syndrome

SUPPLEMENTAL FIGURES

Figure S1. DNAmAge (pan-tissue clock) and age acceleration in newborns with and without Down syndrome.

The different distributions of the DNAmAge epigenetic clock in DS newborns (n = 346) and non-DS newborns (n = 567) are shown as a density plot (panel **A**) and a boxplot (panel **B**). The correlations between DNAmAge and chronological age are shown in scatterplots for DS and non-DS newborns combined (DS n = 294, non-DS n = 541, panel **C**), for DS newborns only (n = 294, panel **D**), and for non-DS newborns only (n = 541, panel **E**). Panel **F** shows the correlation between DNAmAge and chronological age in DS (red, n = 294) and in non-DS newborns (blue, n = 541). The different distributions of DNAmAA (age acceleration using DNAmAge) in DS newborns (n = 294) and non-DS newborns (n = 541) are shown as a density plot (panel **G**) and a boxplot (panel **H**).

P values from the Student t-test are shown in panels **B** and **H**. Spearman correlation coefficient R and its P value of each correlation was summarized in panels **C-F**. The linear trend and its confidence interval of each correlation was summarized in panels **C-E**.

Figure S1

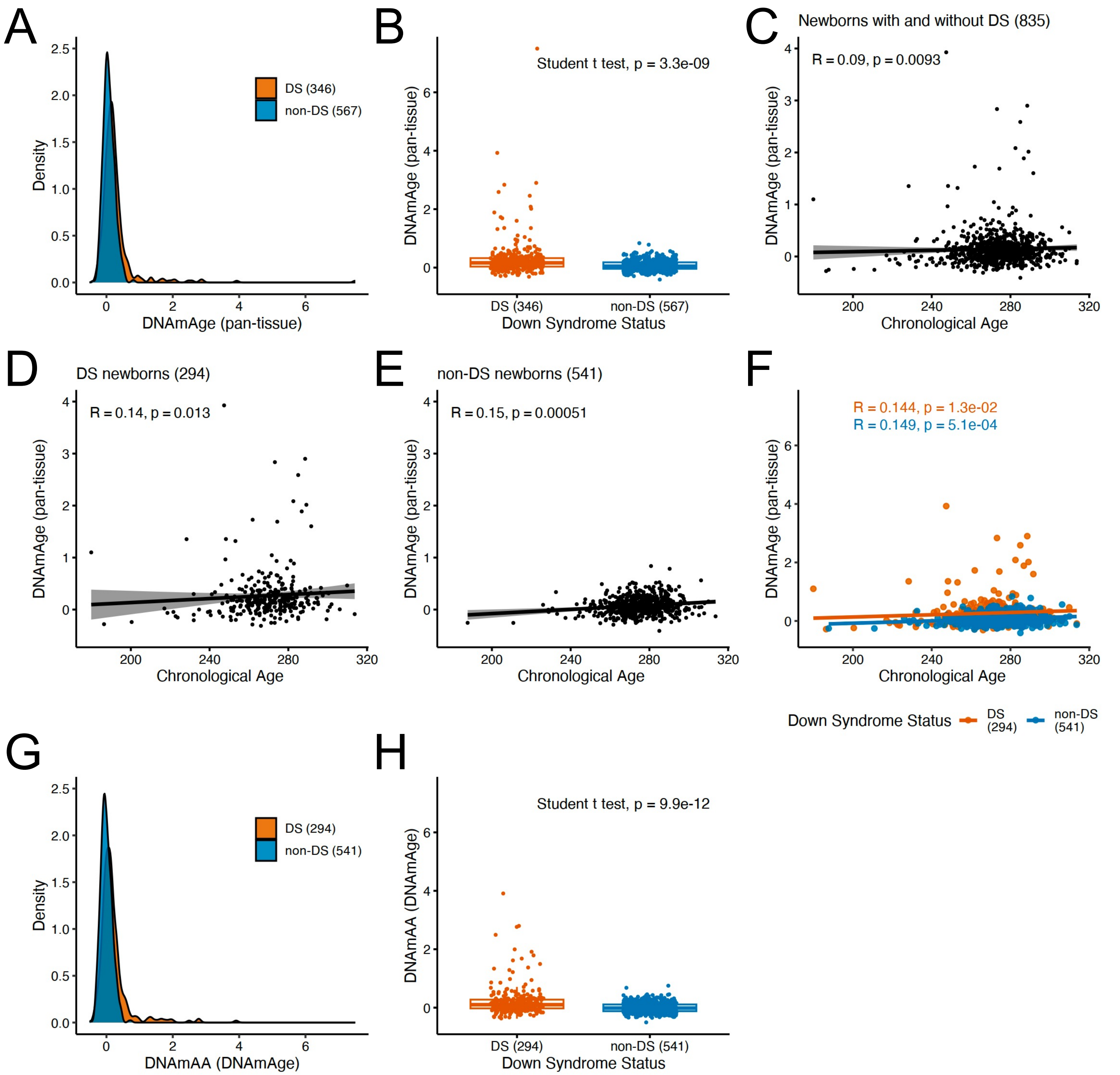

A
Density
DNAmAge (pan-tissue)
DS (346)
non-DS (567)
B
Student t test, p = 3.3e-09
DNAmAge (pan-tissue)
DS (346)
non-DS (567)
Down Syndrome Status
C
Newborns with and without DS (835)
R = 0.09, p = 0.0093
DNAmAge (pan-tissue)
Chronological Age
D
DS newborns (294)
R = 0.14, p = 0.013
DNAmAge (pan-tissue)
Chronological Age
E
non-DS newborns (541)
R = 0.15, p = 0.00051
DNAmAge (pan-tissue)
Chronological Age
F
R = 0.144, p = 1.3e-02
R = 0.149, p = 5.1e-04
DNAmAge (pan-tissue)
Chronological Age
Down Syndrome Status
DS (294)
non-DS (541)
G
Density
DNAmAA (DNAmAge)
DS (294)
non-DS (541)
H
Student t test, p = 9.9e-12
DNAmAA (DNAmAge)
DS (294)
non-DS (541)
Down Syndrome Status

Figure S2. Six newborns with Down syndrome with likely mosaic/partial trisomy 21. The copy number variation (CNV) plots generated by the R package “conumee” for the 6 newborns with likely mosaic/partial trisomy 21 are shown in panels **A-F**. Twenty randomly selected non-DS samples were used to construct a CNV reference. All the 6 DS-newborns appear to have increased copy number on chromosome 21 relative to other chromosomes, but a median chromosome 21 log2 ratio >2 standard deviations below the average median chromosome 21 log2 ratio across all DS newborns. Their median log2 ratios on chromosome 21 as calculated across 317 bins from “conumee” ranged from 0.08 to 0.18. Two randomly selected CNV plots from DS newborns with full T21 are shown in panels **G** and **H** to display the typically higher log2 ratios across chromosome 21.

Figure S2

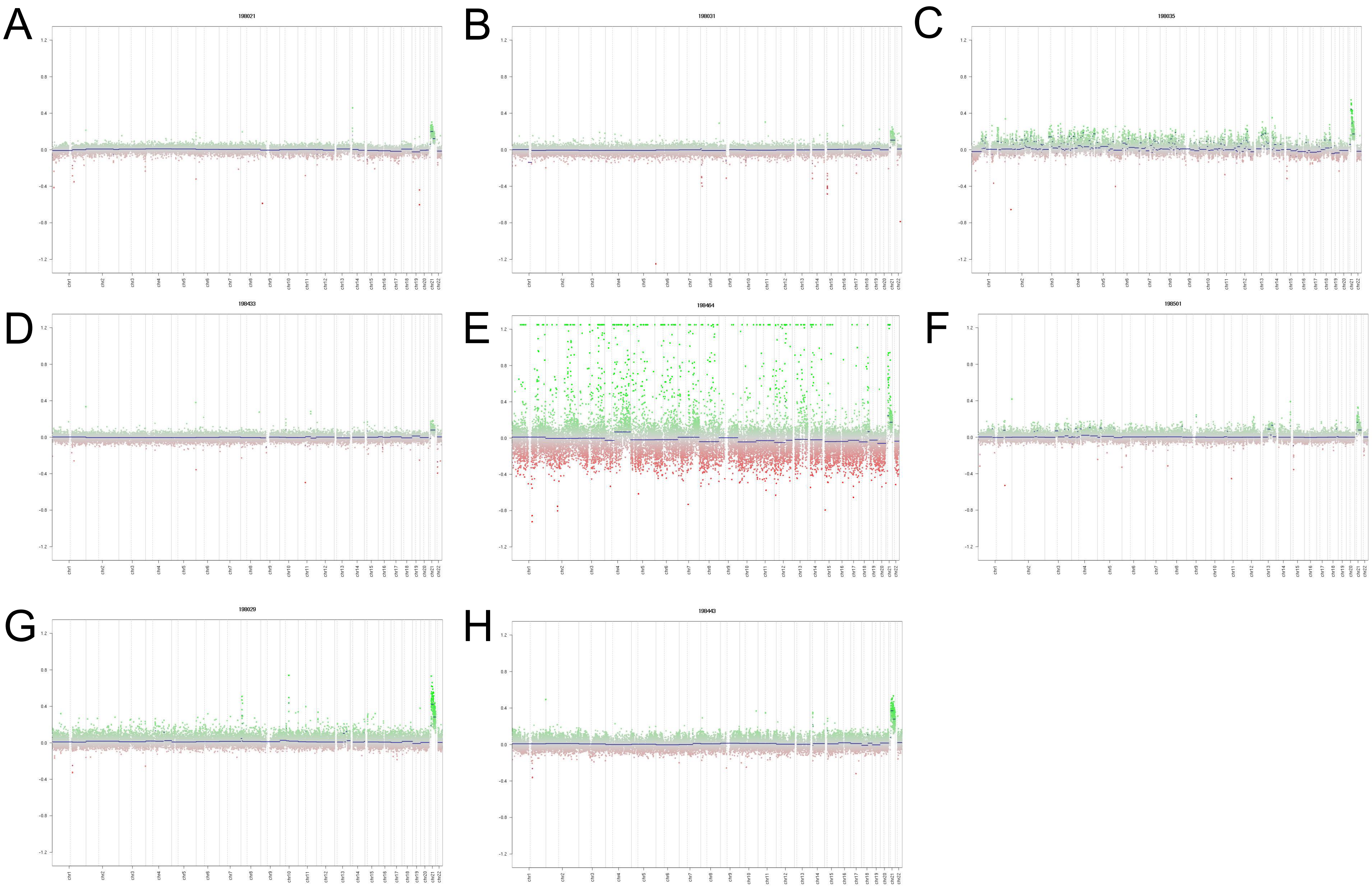
A
198021
B
198031
C
198035
D
198433
E
198464
F
198501
G
198029
H
198443

Figure S3. DNAmAge (pan-tissue clock) and age acceleration in newborns with Down syndrome with full trisomy 21, with likely mosaic/partial trisomy 21 and in newborns without Down syndrome.

The different distributions of the DNAmAge pan-tissue epigenetic clock in full T21 DS newborns (n = 340), likely mosaic/partial T21 DS newborns (n = 6), and non-DS newborns (n = 567) are shown as a box plot (panel **A**). The different distributions of the epigenetic age acceleration (DNAmAA) derived from DNAmAge in full T21 DS newborns (n = 288), likely mosaic/partial T21 DS newborns (n = 6), and non-DS newborns (n = 541) with available birth variable data are shown as a box plot (panel **B**). The global P value from Kruskal-Wallis test and the Benjamini-Hochberg adjusted P values from the pairwise comparison tests using Wilcoxon rank sum test are shown in panels **A and B**. Dots were overlaid on the boxplot to show the individual level data colored by different T21 status.

Figure S3

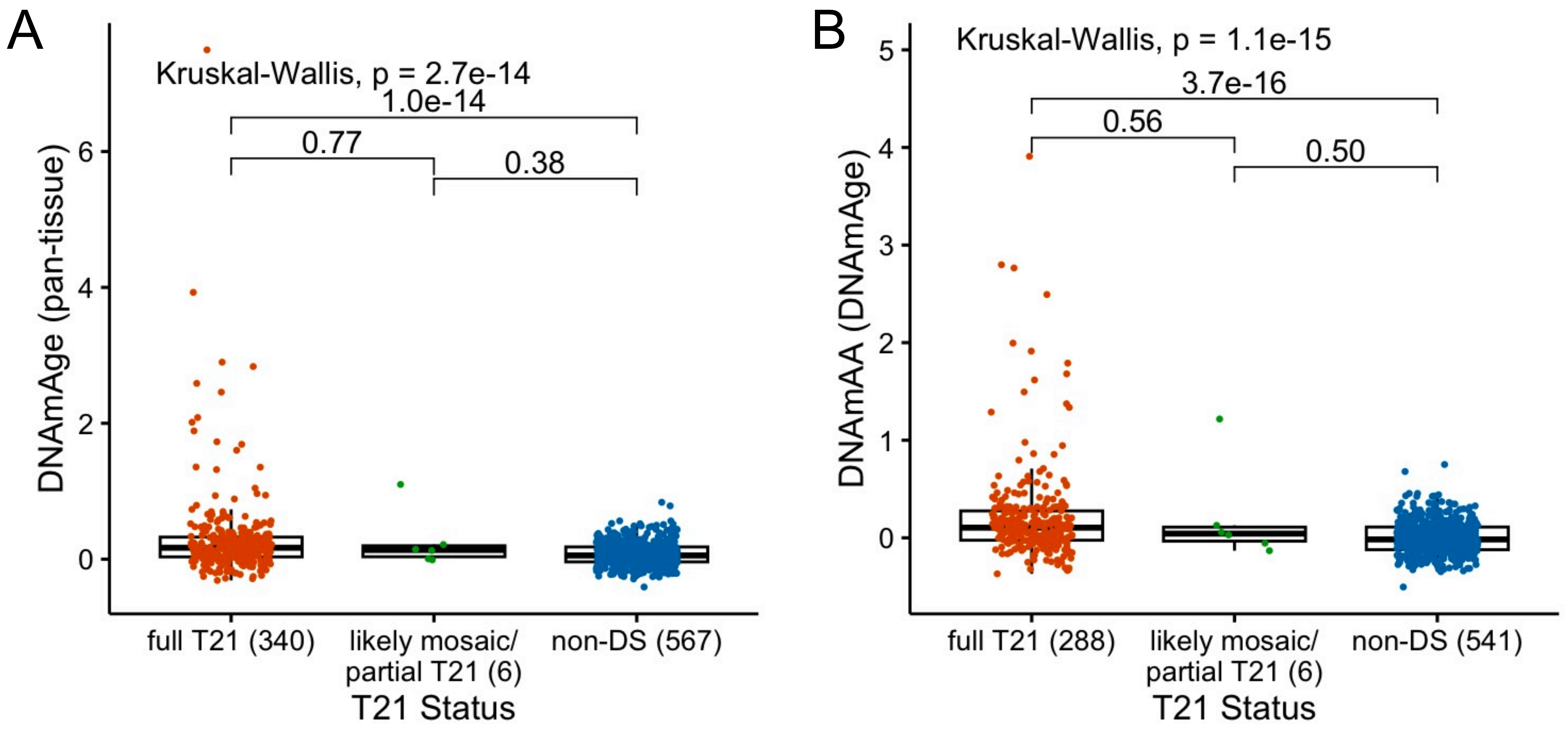
A
Kruskal-Wallis, p = 2.7e-14
1.0e-14
0.77
0.38
DNAmAge (pan-tissue)
6
4
2
0
full T21 (340)
likely mosaic/
partial T21 (6)
non-DS (567)
T21 Status
B
Kruskal-Wallis, p = 1.1e-15
3.7e-16
0.56
0.50
DNAmAA (DNAmAge)
5
4
3
2
1
0
full T21 (288)
likely mosaic/
partial T21 (6)
non-DS (541)
T21 Status

Figure S4. The correlation between DNAmSkinBloodClock and DNAmAge.

The correlation between DNAmSkinBloodClock and DNAmAge are shown in scatterplots for DS and non-DS newborns combined (DS n = 346, non-DS n = 567, panel **A**), for DS newborns only (n = 346, panel **B**), and for non-DS newborns only (n = 567, panel **C**). Spearman correlation coefficient R and its P value, and the linear trend and its confidence interval of each correlation was summarized in panels **A-C**.

Figure S4

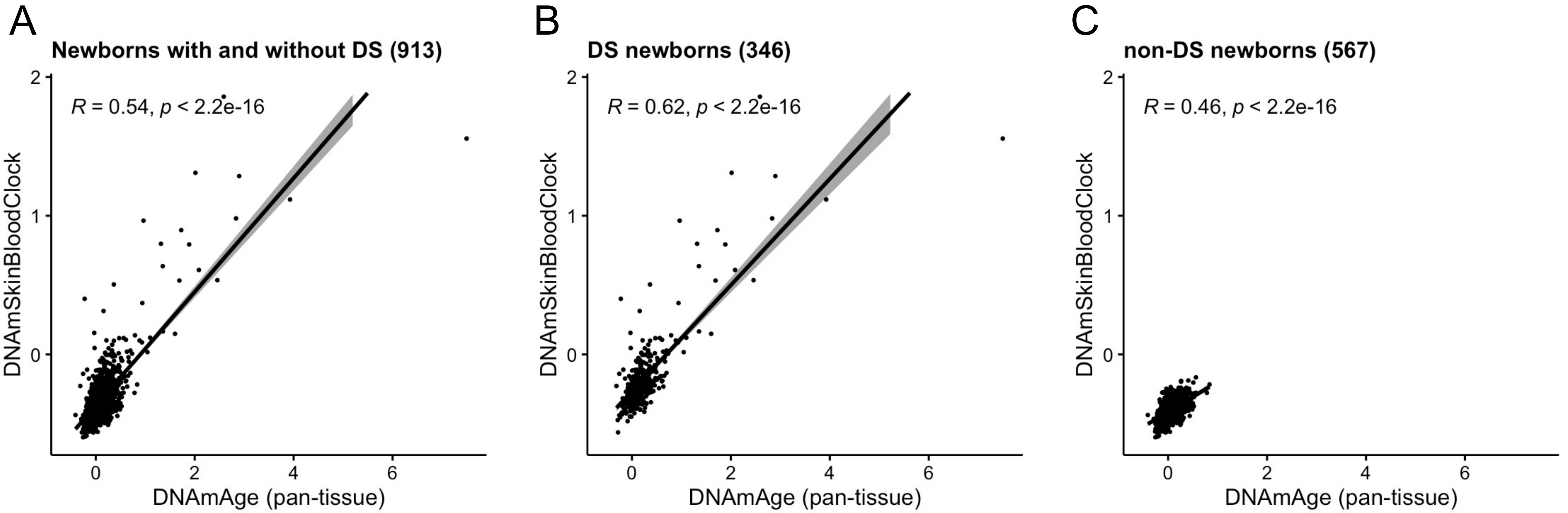
A
Newborns with and without DS (913)
R = 0.54, p < 2.2e-16
DNAmSkinBloodClock
DNAmAge (pan-tissue)
B
DS newborns (346)
R = 0.62, p < 2.2e-16
DNAmSkinBloodClock
DNAmAge (pan-tissue)
C
non-DS newborns (567)
R = 0.46, p < 2.2e-16
DNAmSkinBloodClock
DNAmAge (pan-tissue)

Figure S5. The correlations between the blood cell proportions and DNAmSkinBloodClock in DS (red, n = 346) and non-DS (blue, n = 567) newborns. Spearman correlation coefficient R and its P value, and the Loess smooth curve and its confidence interval of each correlation was summarized in each panel. Bcell B lymphocytes, CD4T CD4+ T lymphocytes, CD8T CD8+ T lymphocytes, Gran granulocytes, Mono monocytes, NK natural killer cells, nRBC nucleated red blood cell.

Figure S5

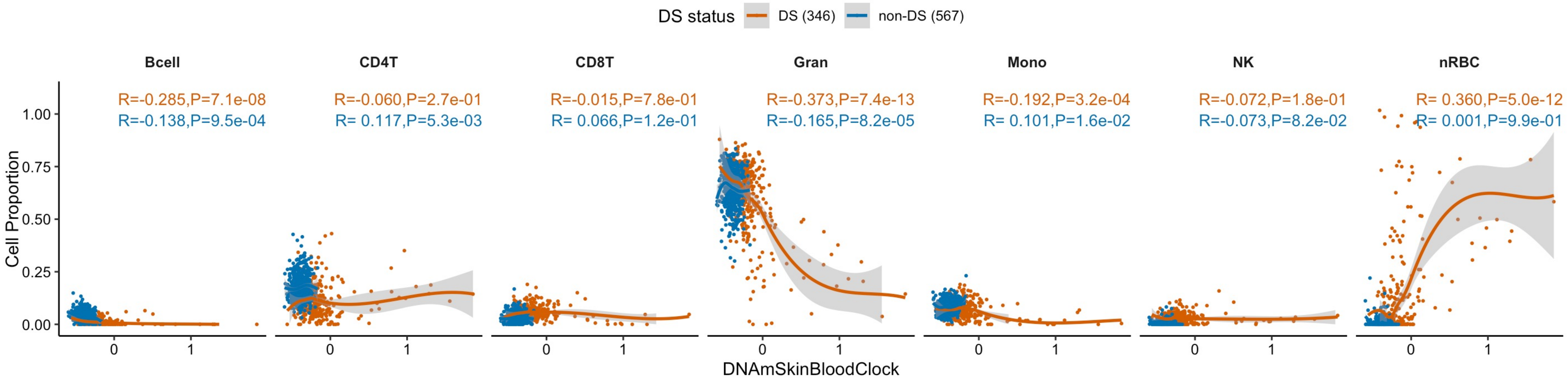

DS status
DS (346)
non-DS (567)
Bcell
CD4T
CD8T
Gran
Mono
NK
nRBC
R=-0.285,P=7.1e-08
R=-0.138,P=9.5e-04
R=-0.060,P=2.7e-01
R= 0.117,P=5.3e-03
R=-0.015,P=7.8e-01
R= 0.066,P=1.2e-01
R=-0.373,P=7.4e-13
R=-0.165,P=8.2e-05
R=-0.192,P=3.2e-04
R= 0.101,P=1.6e-02
R=-0.072,P=1.8e-01
R=-0.073,P=8.2e-02
R= 0.360,P=5.0e-12
R= 0.001,P=9.9e-01
Cell Proportion
1.00
0.75
0.50
0.25
0.00
0
1
DNAmSkinBloodClock

Figure S6. Epigenetic age and age acceleration in Down syndrome newborns with and without acute lymphoblastic leukemia.

The different distributions of the DNAmSkinBloodClock epigenetic clock in DS newborns that later developed ALL (DS-ALL, n = 147) and DS newborns without ALL (DS controls, n = 199) are shown as a density plot (panel **A**) and a boxplot (panel **B**). The different distributions of the DNAmAge epigenetic clock in DS-ALL cases (n = 147) and DS controls (n = 199) are shown as a density plot (panel **C**) and a boxplot (panel **D**). The different distributions of DNAmAA derived from DNAmSkinBloodClock in DS-ALL cases (n = 115) and DS controls (n = 179) are shown as a density plot (panel **E**) and a boxplot (panel **F**). The different distributions of DNAmAA derived from DNAmAge in DS-ALL cases (n = 115) and DS controls (n = 179) are shown as a density plot (panel **G**) and a boxplot (panel **H**). P values from the Student t-test are shown in panels **B**, **D, F** and **H**.

Figure S6

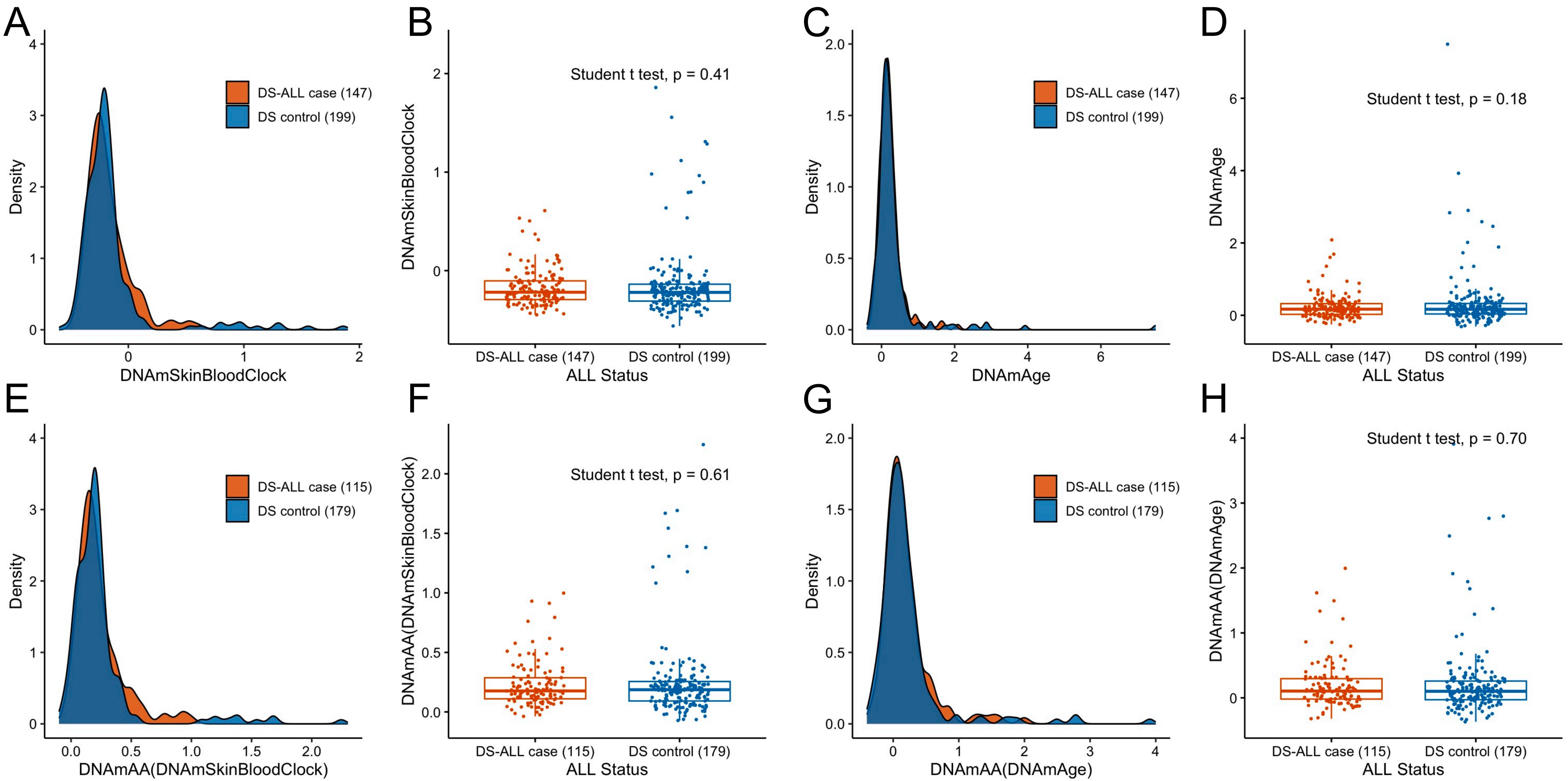

A
B
C
D
E
F
G
H
Density
DNAmSkinBloodClock
DS-ALL case (147)
DS control (199)
Student t test, p = 0.41
ALL Status
DNAmAge
Student t test, p = 0.18
DNAmAA(DNAmSkinBloodClock)
DS-ALL case (115)
DS control (179)
Student t test, p = 0.61
DNAmAA(DNAmAge)
Student t test, p = 0.70

Figure S7. Epigenetic age and age acceleration in Down syndrome newborns with *GATA1* mutation and without *GATA1* mutation.

The different distributions of the DNAmSkinBloodClock epigenetic clock in DS newborns with *GATA1* mutation (n = 30) and DS newborns without *GATA1* mutation (n = 154) are shown as a density plot (panel **A**) and a boxplot (panel **B**). The different distributions of DNAmAA (DNAmSkinBloodClock) in DS newborns with *GATA1* mutation (n = 26) and DS newborns without *GATA1* mutation (n = 139) are shown as a density plot (panel **C**) and a boxplot (panel **D**). P values from the Student t-test are shown in panels **B**, and **D**.

Figure S7

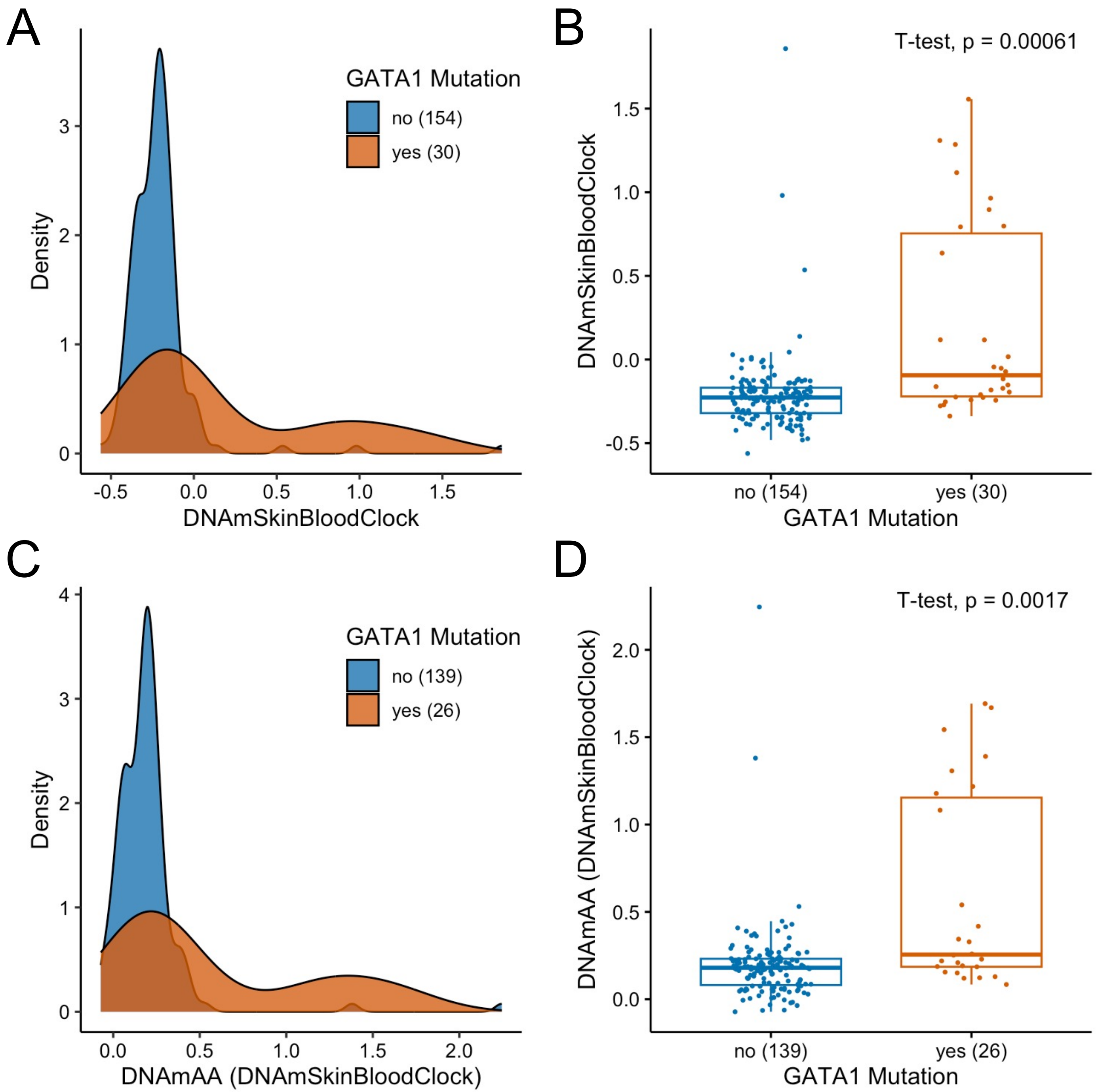
A
GATA1 Mutation
no (154)
yes (30)
Density
DNAmSkinBloodClock
-0.5 0.0 0.5 1.0 1.5
0 1 2 3
B
T-test, p = 0.00061
DNAmSkinBloodClock
-0.5 0.0 0.5 1.0 1.5
no (154) yes (30)
GATA1 Mutation
C
GATA1 Mutation
no (139)
yes (26)
Density
0 1 2 3 4
0.0 0.5 1.0 1.5 2.0
DNAmAA (DNAmSkinBloodClock)
D
T-test, p = 0.0017
DNAmAA (DNAmSkinBloodClock)
0.0 0.5 1.0 1.5 2.0
no (139) yes (26)
GATA1 Mutation

Figure S8. The correlations between the Haftorn, Knight, and Bohlin clocks in DS and non-DS newborns combined (n = 913), in DS newborns (n = 346), and in non-DS newborns (n = 567).

The correlations between the Haftorn and Knight clocks are shown in scatterplots for DS and non-DS newborns combined (panel **A**), for DS newborns only (panel **B**), and for non-DS newborns only (panel **C**). The correlations between the Haftorn and Bohlin clocks are shown in scatterplots for DS and non-DS newborns combined (panel **D**), for DS newborns only (panel **E**), and for non-DS newborns only (panel **F**). The correlations between the Knight and Bohlin clocks are shown in scatterplots for DS and non-DS newborns combined (panel **G**), for DS newborns only (panel **H**), and for non-DS newborns only (panel **I**). Spearman correlation coefficient R and its P value, and the linear trend and its confidence interval of each correlation was summarized in each panel.

Figure S8

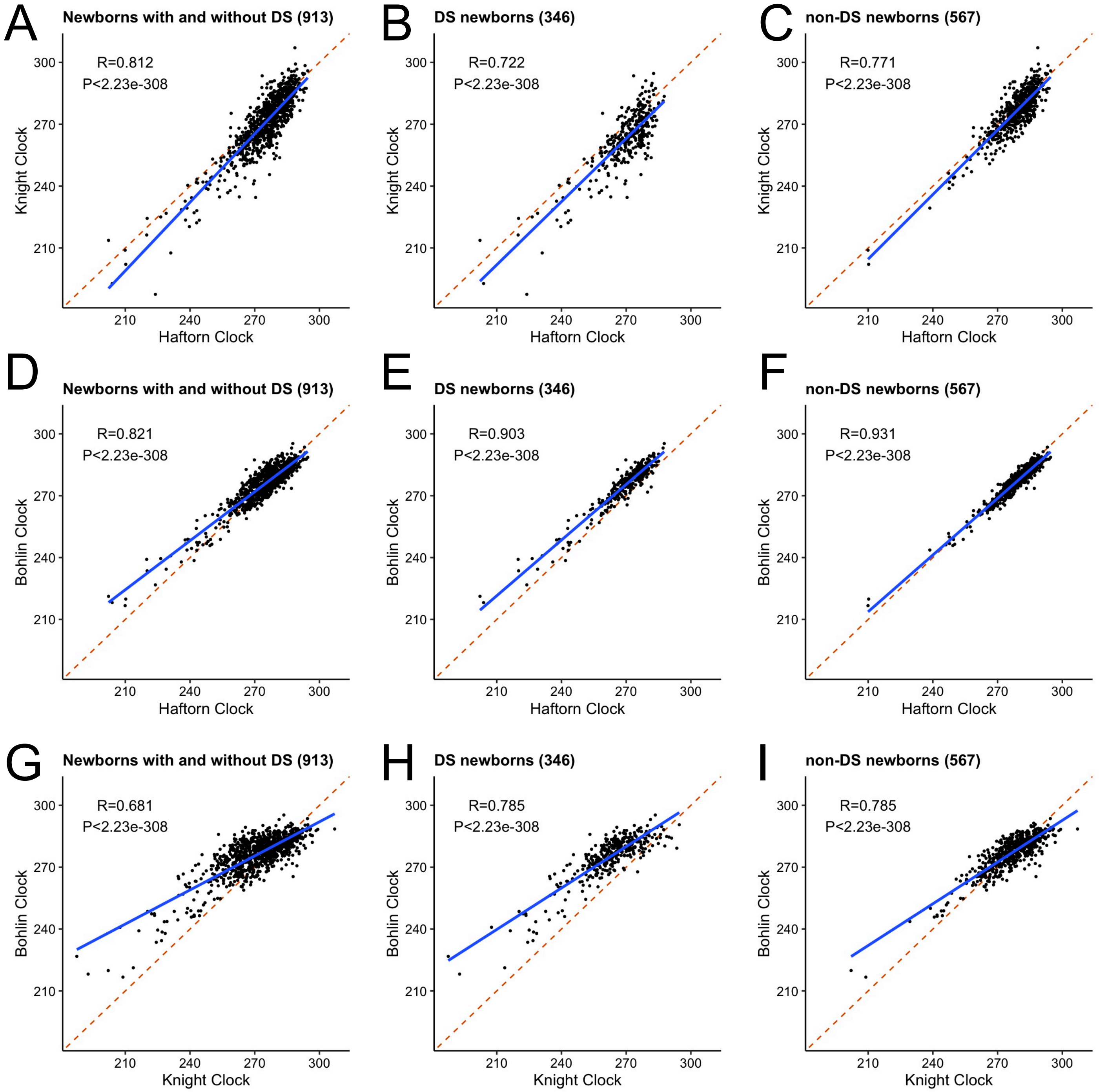

A
Newborns with and without DS (913)
R=0.812
P<2.23e-308
Knight Clock
Haftorn Clock
B
DS newborns (346)
R=0.722
P<2.23e-308
Knight Clock
Haftorn Clock
C
non-DS newborns (567)
R=0.771
P<2.23e-308
Knight Clock
Haftorn Clock
D
Newborns with and without DS (913)
R=0.821
P<2.23e-308
Bohlin Clock
Haftorn Clock
E
DS newborns (346)
R=0.903
P<2.23e-308
Bohlin Clock
Haftorn Clock
F
non-DS newborns (567)
R=0.931
P<2.23e-308
Bohlin Clock
Haftorn Clock
G
Newborns with and without DS (913)
R=0.681
P<2.23e-308
Bohlin Clock
Knight Clock
H
DS newborns (346)
R=0.785
P<2.23e-308
Bohlin Clock
Knight Clock
I
non-DS newborns (567)
R=0.785
P<2.23e-308
Bohlin Clock
Knight Clock

Figure S9. The correlations between the three epigenetic gestational age clocks and the observed gestational age in DS and non-DS newborns combined (n = 847), in DS newborns (n = 306), and in non-DS newborns (n = 541).

The correlations between the Haftorn clock and gestational age are shown in scatterplots for DS and non-DS newborns combined (panel **A**), for DS newborns only (panel **B**), and for non-DS newborns only (panel **C**). The correlations between the Knight clock and gestational age are shown in scatterplots for DS and non-DS newborns combined (panel **D**), for DS newborns only (panel **E**), and for non-DS newborns only (panel **F**). The correlations between the Bohlin clock and gestational age are shown in scatterplots for DS and non-DS newborns combined (panel **G**), for DS newborns only (panel **H**), and for non-DS newborns only (panel **I**). Spearman correlation coefficient R and its P value, and the linear trend and its confidence interval of each correlation was summarized in each panel.

Figure S9

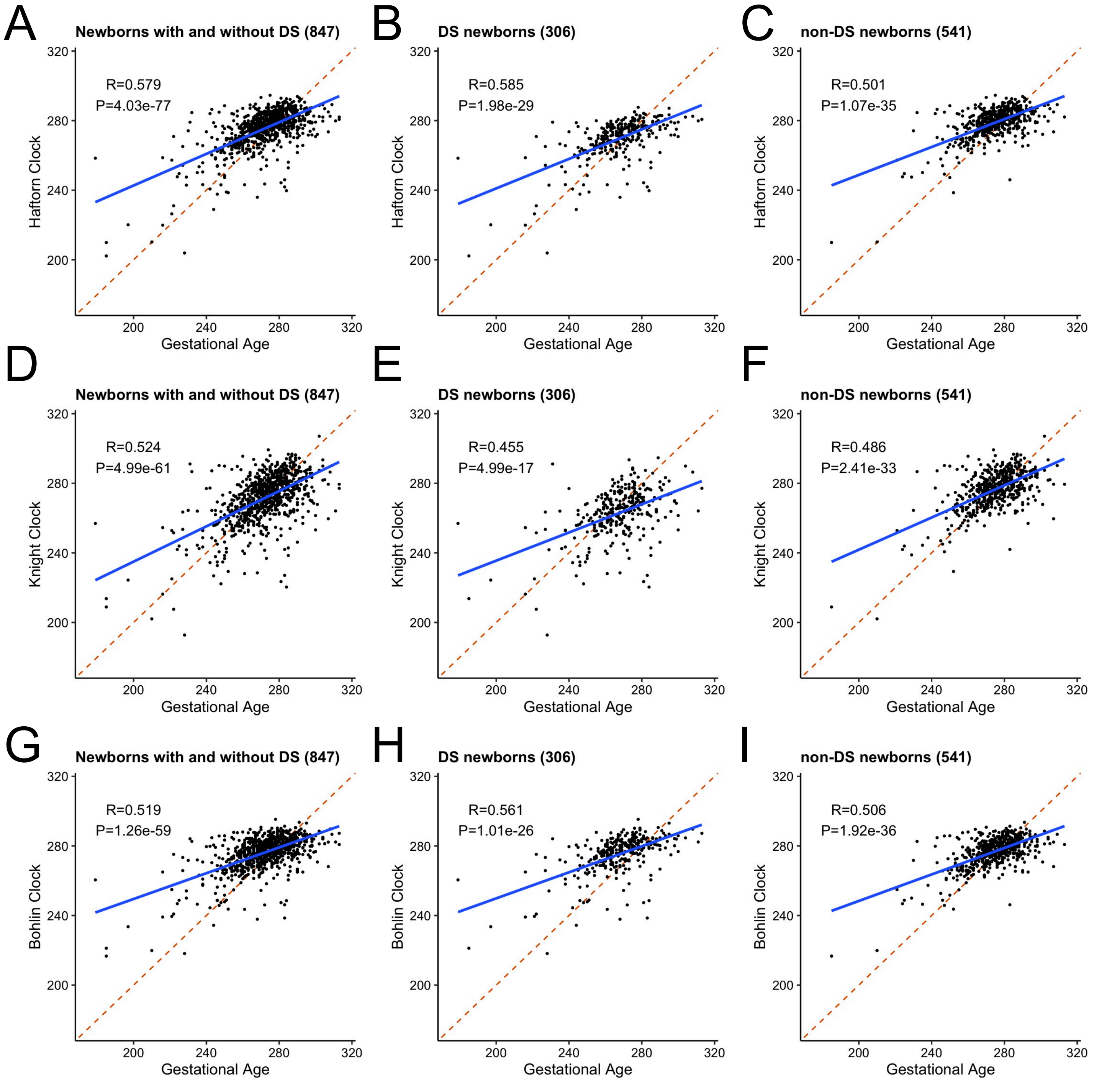
A
Newborns with and without DS (847)
R=0.579
P=4.03e-77
Hafforn Clock
Gestational Age
B
DS newborns (306)
R=0.585
P=1.98e-29
Hafforn Clock
Gestational Age
C
non-DS newborns (541)
R=0.501
P=1.07e-35
Hafforn Clock
Gestational Age
D
Newborns with and without DS (847)
R=0.524
P=4.99e-61
Knight Clock
Gestational Age
E
DS newborns (306)
R=0.455
P=4.99e-17
Knight Clock
Gestational Age
F
non-DS newborns (541)
R=0.486
P=2.41e-33
Knight Clock
Gestational Age
G
Newborns with and without DS (847)
R=0.519
P=1.26e-59
Bohlin Clock
Gestational Age
H
DS newborns (306)
R=0.561
P=1.01e-26
Bohlin Clock
Gestational Age
I
non-DS newborns (541)
R=0.506
P=1.92e-36
Bohlin Clock
Gestational Age
200
240
280
320

Figure S10. The observed gestational age and the three gestational epigenetic age clocks in newborns with and without Down syndrome. The different distributions of the observed gestational age in DS newborns (n = 306) and non-DS newborns (n = 541) are shown as a density plot (panel **A**) and a boxplot (panel **B**). The different distributions of the Haftorn clock in DS newborns (n = 346) and non-DS newborns (n = 567) are shown as a density plot (panel **C**) and a boxplot (panel **D**). The different distributions of the Knight clock in DS newborns (n = 346) and non-DS newborns (n = 567) are shown as a density plot (panel **E**) and a boxplot (panel **F**). The different distributions of the Bohlin clock in DS newborns (n = 346) and non-DS newborns (n = 567) are shown as a density plot (panel **G**) and a boxplot (panel **H**). P values from the Student t-test are shown in panels **B**, **D, F** and **H**.

Figure S10

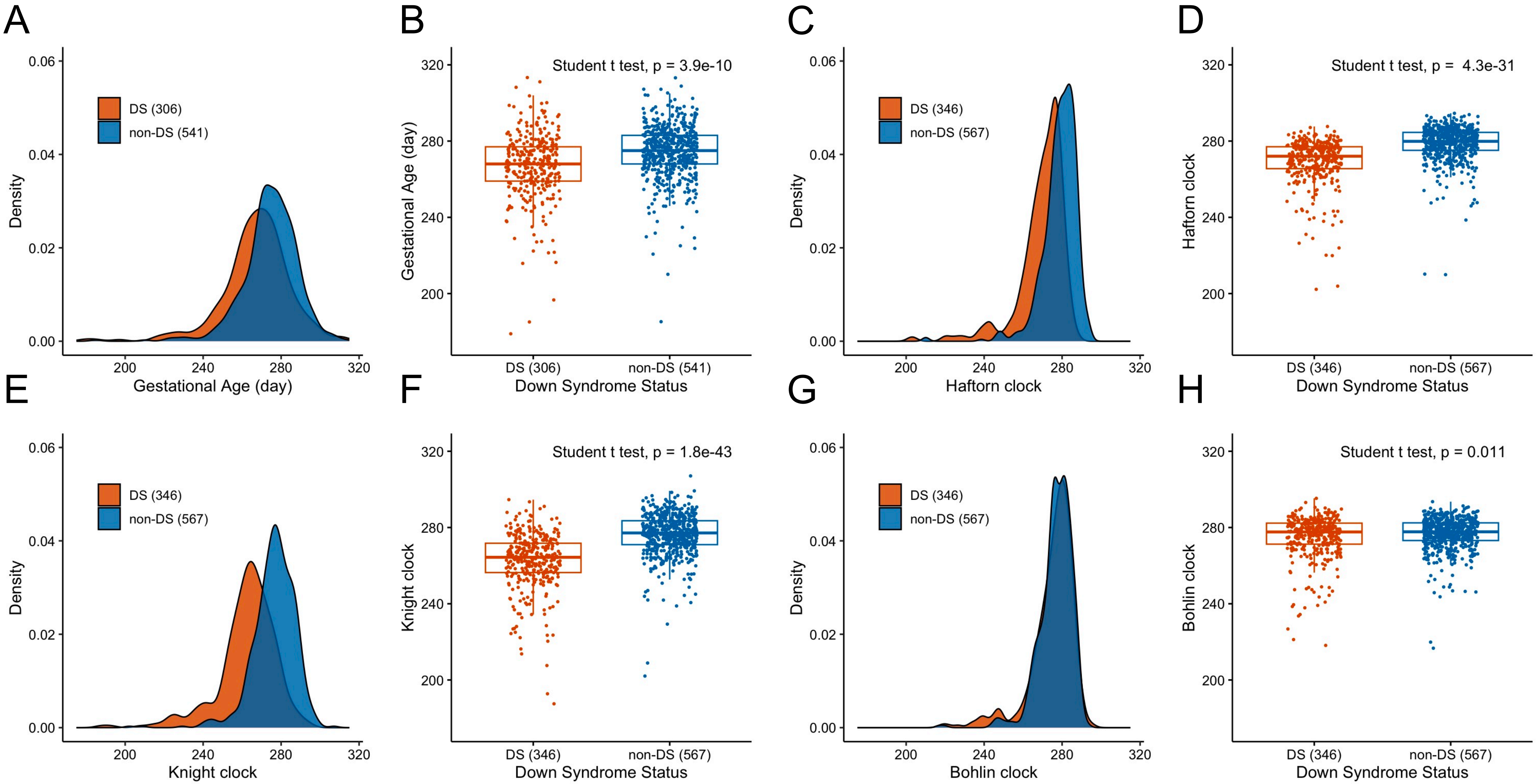
A
0.06
0.04
0.02
0.00
Density
DS (306)
non-DS (541)
200
240
280
320
Gestational Age (day)
B
Student t test, p = 3.9e-10
320
280
240
200
Gestational Age (day)
DS (306)
non-DS (541)
Down Syndrome Status
C
Density
DS (346)
non-DS (567)
Haftorn clock
D
Student t test, p = 4.3e-31
Haftorn clock
DS (346)
non-DS (567)
Down Syndrome Status
E
Density
DS (346)
non-DS (567)
Knight clock
F
Student t test, p = 1.8e-43
Knight clock
DS (346)
non-DS (567)
Down Syndrome Status
G
Density
DS (346)
non-DS (567)
Bohlin clock
H
Student t test, p = 0.011
Bohlin clock
DS (346)
non-DS (567)
Down Syndrome Status

Figure S11. DNAmAA derived from the three epigenetic gestational age clocks in DS newborns (n = 306) and non-DS newborns (n = 541).

The different distributions of DNAmAA derived from the Haftorn clock in DS newborns and non-DS newborns are shown as a density plot (panel **A**) and a boxplot (panel **B**). The different distributions of DNAmAA derived from the Knight clock in DS newborns and non-DS newborns are shown as a density plot (panel **C**) and a boxplot (panel **D**). The different distributions of DNAmAA derived from the Bohlin clock in DS newborns and non-DS newborns are shown as a density plot (panel **E**) and a boxplot (panel **F**). P values from the Student t-test are shown in panels **B**, **D,** and **F**.

Figure S11

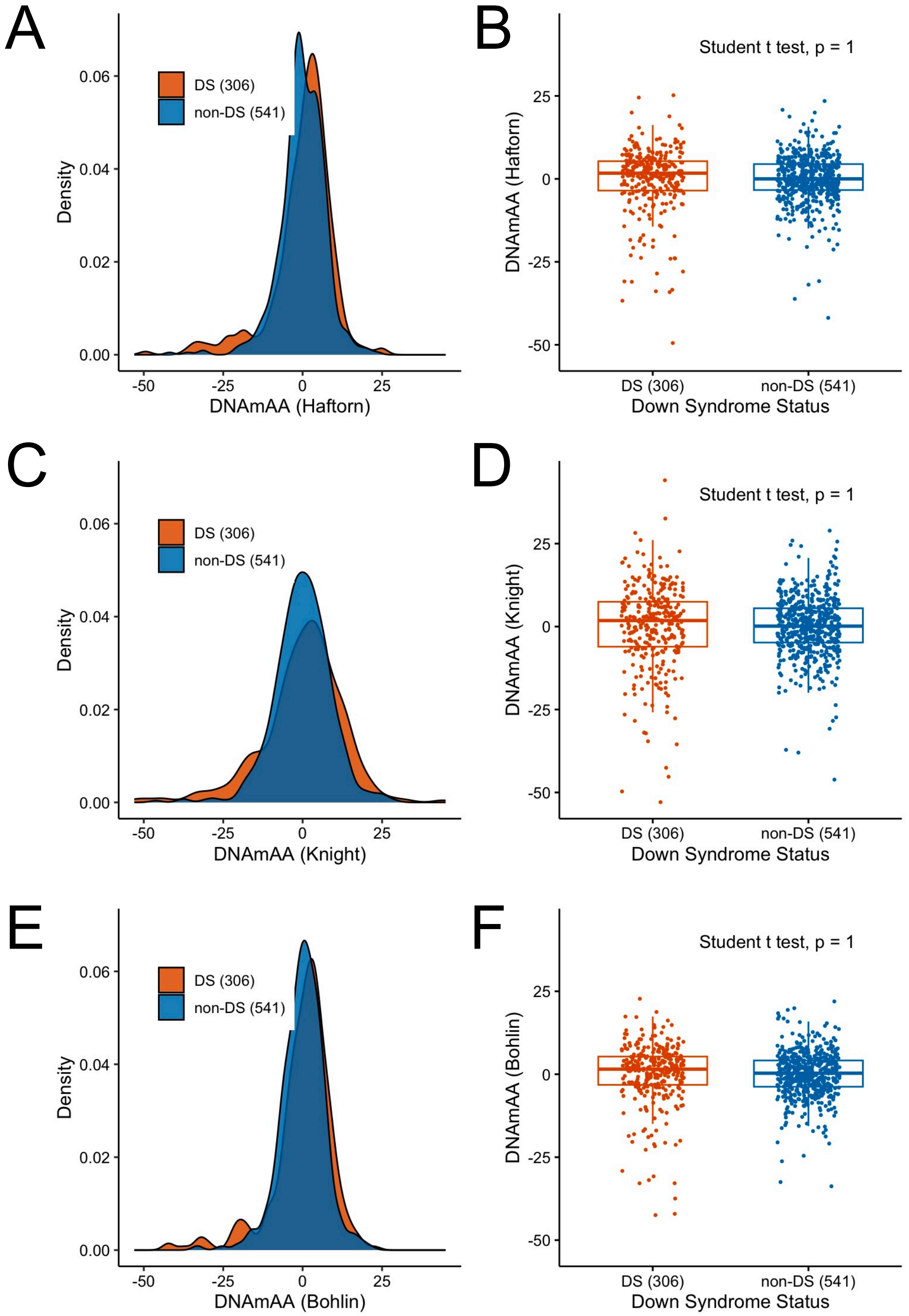

A
B
C
D
E
F
DS (306)
non-DS (541)
Density
0.06
0.04
0.02
0.00
-50
-25
0
25
DNAmAA (Haftorn)
DNAmAA (Knight)
DNAmAA (Bohlin)
Student t test, p = 1
-50
-25
0
25
DS (306)
non-DS (541)
Down Syndrome Status

Figure S12. The number of the CpGs overlapping each pair of the epigenetic clocks (i.e., DNAmSkinBloodClock, DNAmAge, Haftorn clock, Knight clock, and Bohlin clock).

Figure S12

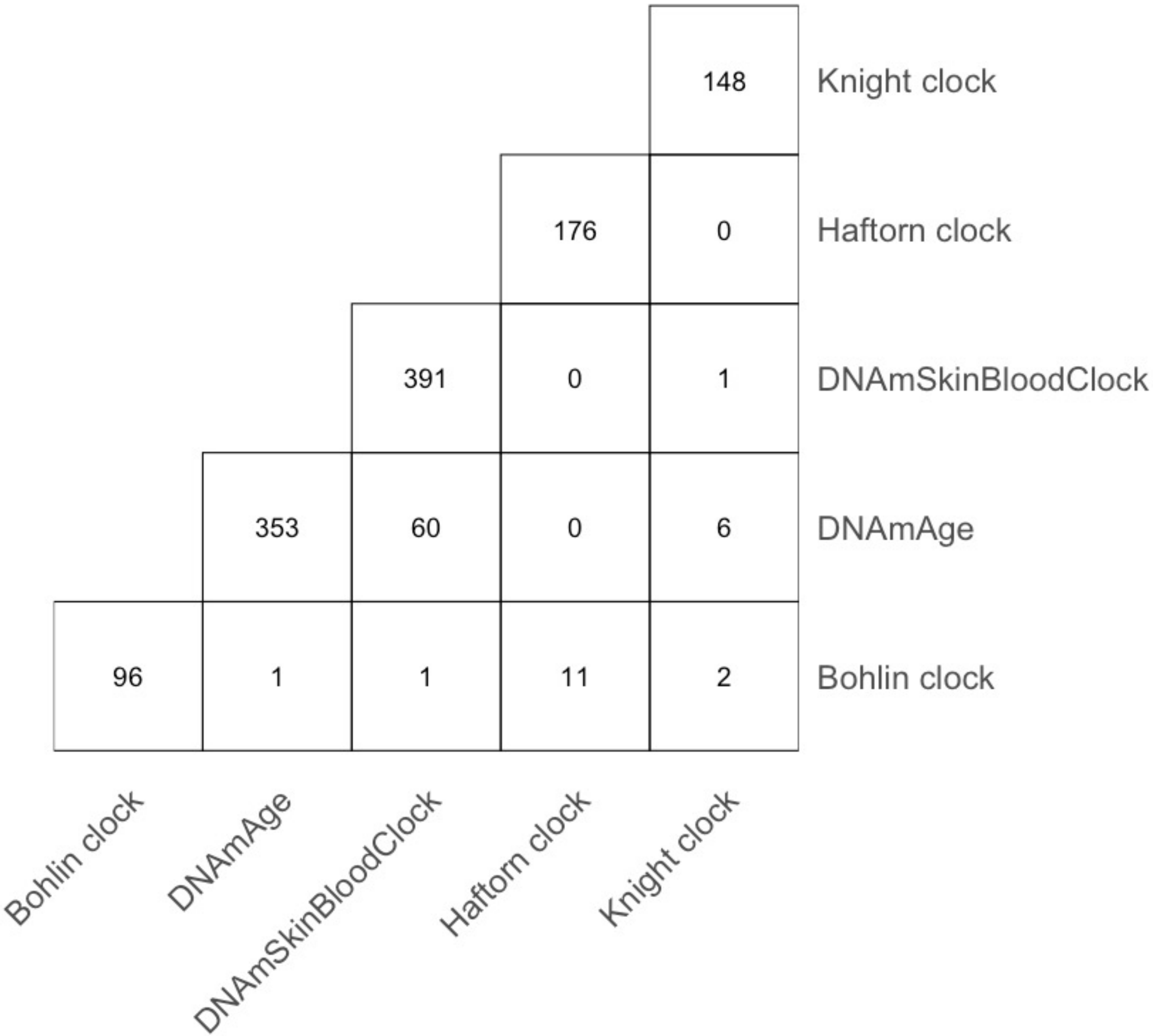
148
Knight clock
176
0
Haftorn clock
391
0
1
DNAmSkinBloodClock
353
60
0
6
DNAmAge
96
1
1
11
2
Bohlin clock
Bohlin clock
DNAmAge
DNAmSkinBloodClock
Haftorn clock
Knight clock
